# Tailoring Multifunctional Carbon Dots via Precursor Stoichiometry: Switching Between Solid‐State Fluorescence and Broadband Absorption Through Aggregation Control

**DOI:** 10.1002/advs.75737

**Published:** 2026-05-15

**Authors:** Gaixia Yang, Hao Sun, Defa Hou, Fulin Yang, Yuan Zou, Bei Zhou, Lanxiang Liu, Xu Lin, Guanben Du

**Affiliations:** ^1^ National Joint Engineering Research Center for Highly‐Efficient Utilization Technology of Forestry Resources Southwest Forestry University Kunming Yunnan China; ^2^ Yunnan Key Laboratory of Breeding and Utilization of Resource Insects Key Laboratory of Protection and Utilization of Insects (National Forestry and Grassland Administration) Research Center of Engineering and Technology of Characteristic Forest Resources (National Forestry and Grassland Administration) Institute of Highland Forest Science Chinese Academy of Forestry Kunming China

**Keywords:** aggregation control, carbon dots, hydrogen bonding, surface chemistry, *π*–*π* stacking

## Abstract

Achieving precise control over the solid‐state optical properties of carbon dots (CDs) remains a significant challenge, primarily due to aggregation‐caused quenching (ACQ) and their intrinsically narrow absorption. Herein, we establish a framework that links surface chemistry to aggregation behavior by demonstrating that precursor stoichiometry programs the surface functionality of CDs, which in turn dictates their packing geometry. By varying the molar ratio of 2,3‐diaminonaphthalene to *o*‐phthalaldehyde, CDs with distinct surface chemistries are engineered. Under aldehyde‐rich conditions, CDs with aldehyde‐enriched surfaces self‐assemble into ordered, layered structures through directional hydrogen bonding. This ordered arrangement effectively suppresses *π*–*π* stacking and enables efficient red solid‐state fluorescence (SSF). Conversely, amine‐rich surfaces promote face‐to‐face *π*–*π* stacking, leading to compact, spherical aggregates. Such dense packing establishes a continuous donor–acceptor interface, facilitating broadband visible absorption through efficient intermolecular charge transfer (ICT). This work establishes a general design principle according to which aldehyde‐rich surfaces enable SSF, while amine‐rich surfaces confer broadband absorption and photothermal capability. Our findings demonstrate that precursor stoichiometry serves as a powerful tool for directing CDs aggregation and provides a unified platform for developing multifunctional CDs‐based materials.

## Graphical abstract

Precursor stoichiometry programs the surface chemistry of carbon dots, directing their self‐assembly into distinct aggregation modes. Aldehyde‐enriched surfaces form ordered, hydrogen‐bonded assemblies that enable bright red solid‐state fluorescence, whereas amine‐rich surfaces promote compact *π*–*π* stacked aggregates with broadband visible‐light absorption. This work establishes a surface‐directed strategy for designing multifunctional solid‐state carbon dot materials.

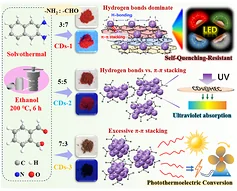

## 1 Introduction

Carbon dots (CDs), generally defined as carbon‐based nanoparticles with diameters below 10 nm, have emerged as a promising class of luminescent nanomaterials owing to their favorable environmental benignity and biocompatibility relative to conventional organic fluorophores and metal‐based emitters [1, 2, 3]. These attributes have stimulated extensive investigations into their use in light‐emitting diodes (LEDs), anti‐counterfeiting, bioimaging, UV shielding, and photothermal conversion [4, 5, 6, 7, 8, 9, 10]. For solid‐state applications, the optical properties of CDs are determined by their aggregation patterns. However, achieving precise control over this aggregation behavior remains a fundamental challenge. Two persistent obstacles, aggregation‐caused quenching (ACQ) and inherent narrow absorption, are manifestations of uncontrolled molecular packing [11, 12, 13]. Therefore, establishing a reasonable framework to guide the aggregation of CDs is crucial for fully exploiting their potential in solid‐state devices.

The optical properties of materials in the solid state are fundamentally governed by their molecular packing patterns. In particular, chromophores can organize into distinct stacking configurations that yield dramatically different photophysical behaviors [14]. One archetypal mode involves a face‐to‐face arrangement where the close proximity of aromatic planes strengthens excitonic coupling and *π*–*π* interactions. This often leads to pronounced fluorescence quenching in the solid state but also facilitates the formation of extended donor–acceptor interfaces that can promote intermolecular charge transfer (ICT) [15, 16]. Such ICT introduces additional low‐energy excitation channels and can extend light absorption toward longer wavelengths. In contrast, a staggered or head‐to‐tail packing mode favors the formation of allowed low‐energy excitonic transitions, typically manifested as red‐shifted absorption and emission. More importantly, this slipped geometry spatially suppresses direct face‐to‐face *π*–*π* contacts, thereby mitigating non‐radiative energy dissipation and quenching associated with tight packing and enabling efficient solid‐state fluorescence (SSF). Given these distinct photophysical outcomes, achieving controllable packing is crucial for realizing targeted optical functions. Accordingly, rationally directing the packing of CDs toward either staggered or face‐to‐face arrangements could provide a pathway to simultaneously overcome ACQ while enabling broadband and/or long‐wavelength absorption. However, precise control over CD aggregation using simple and general synthetic handles remains challenging. Motivated by the central role of packing, we hypothesize that programming CDs aggregation via surface‐chemistry engineering may provide a versatile strategy to address both ACQ and long‐wavelength absorption in a single design framework.

**FIGURE 1:**
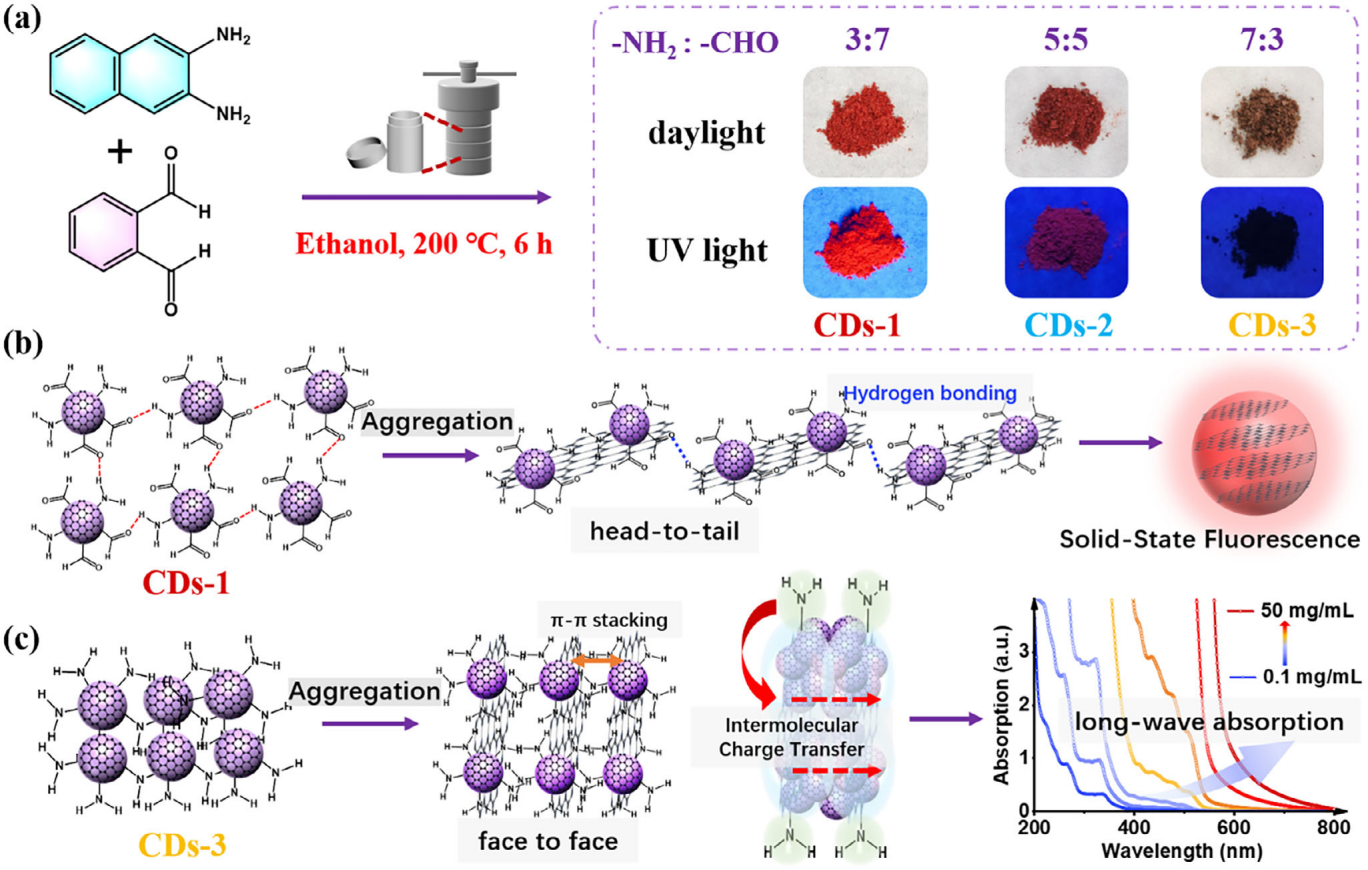
(a) Schematic diagram of the synthesis method of CDs. The mechanism diagram of the aggregation of (b) CDs‐1 and (c) CDs‐3.

Given the critical role of molecular packing, we reasoned that programming the aggregation behavior of CDs by tailoring their surface chemistry could represent a versatile strategy to simultaneously address ACQ and long‐wavelength absorption. However, a fundamental question remains: can the aggregation mode of CDs be rationally directed by engineering their surface functional groups, and if so, what is the underlying mechanism linking surface chemistry to packing geometry? Herein, we address this question by systematically varying the molar ratio of 2,3‐diaminonaphthalene to *o*‐phthalaldehyde, thereby engineering CDs with distinct surface chemistries. CDs with aldehyde‐rich surfaces form ordered, layered assemblies stabilized by hydrogen bonding, achieving efficient SSF. Conversely, CDs with amine‐rich surfaces promote the formation of compact assemblies dominated by face‐to‐face *π*–*π* stacking. The electron‐rich amine groups establish an ideal donor–acceptor interface with the electron‐deficient cores of adjacent CDs, enabling efficient ICT and extending the absorption range into the visible region. CDs with a balanced ratio exhibit intermediate properties. This approach allows us to establish a direct correlation between surface functionality, aggregation pathway, and the resulting optical properties. Our findings provide a general design principle for programming CDs aggregation and offer a unified platform for developing multifunctional CD‐based materials (Figure 1).

## 2 Results and Discussion

### 2.1 Structural Characterization Revealing Surface Chemistry

**FIGURE 2:**
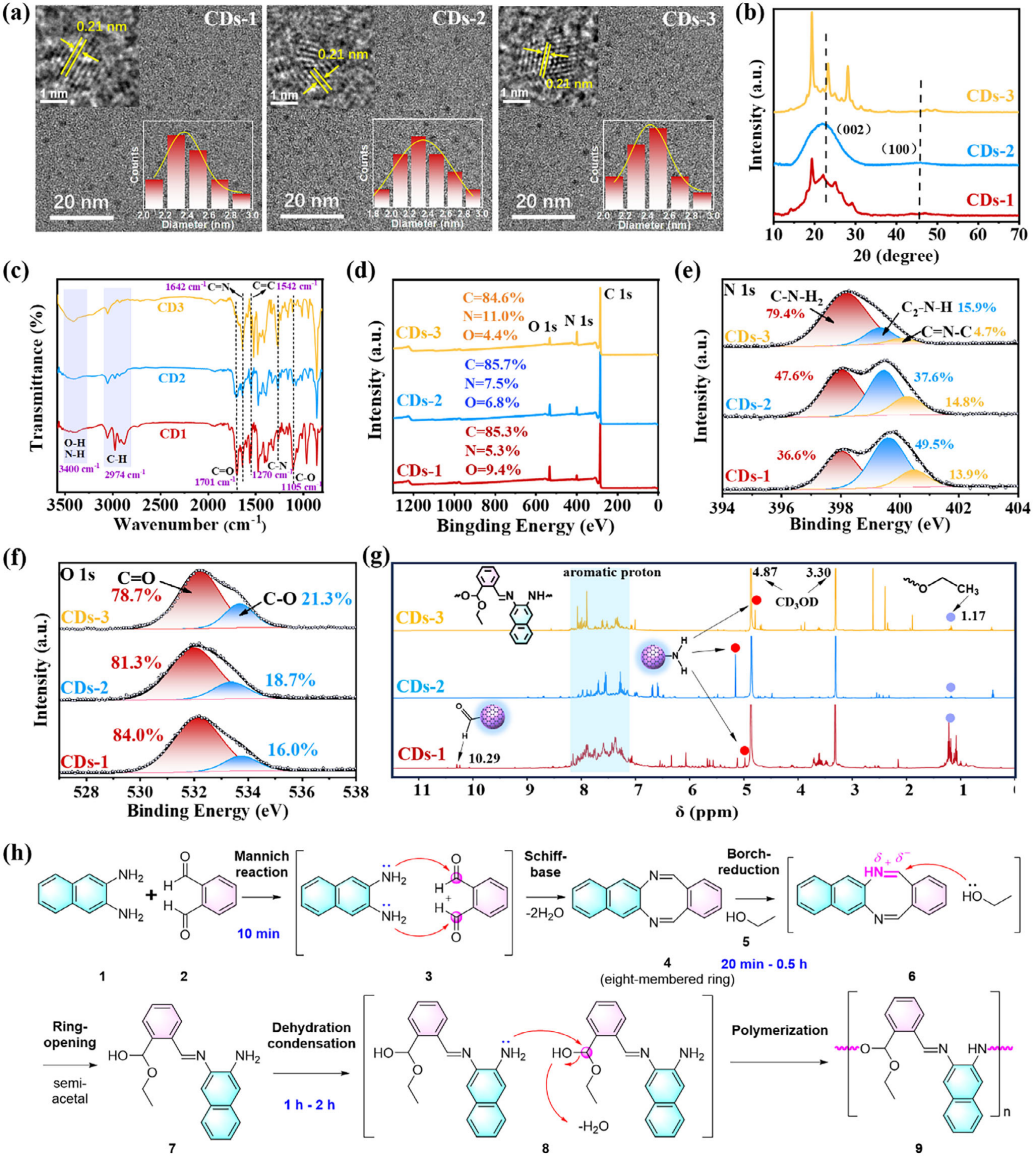
(a) TEM images of CDs‐1, CDs‐2, and CDs‐3, lattice spacing, and histograms of grain size distributions. (b) XRD pattern and (c) FT‐IR spectra of CDs‐1, CDs‐2, and CDs‐3. XPS spectra of CDs‐1, CDs‐2, and CDs‐3: (d) survey scans, (e) high‐resolution N 1s regions, and (f) high‐resolution O 1s regions. (g) ^1^H NMR spectra in CD_3_OD of CDs‐1, CDs‐2, and CDs‐3. (h) Schematic diagram of the synthesis process of CDs.

To elucidate the morphological and chemical differences among the three CD samples, we carried out a comprehensive characterization using transmission electron microscopy (TEM), X‐ray diffraction (XRD), Raman, Fourier transform infrared spectroscopy (FT‐IR), X‐ray photoelectron spectroscopy (XPS), and proton nuclear magnetic resonance (^1^H NMR). TEM images (Figure 2a) showed that three CDs possess highly uniform spherical structures with similar sizes (CDs‐1: 2.3 ± 0.05 nm, CDs‐2: 2.3 ± 0.03 nm, CDs‐3: 2.5 ± 0.03 nm), indicating precursor ratio minimally affects CD dimensions. High‐resolution TEM revealed lattice fringes with 0.21 nm spacing, corresponding to graphite carbon (100) planes [17, 18]. The XRD patterns reveal that CDs‐2 exhibits characteristic features of amorphous carbon, whereas both CDs‐1 and CDs‐3 show sharp diffraction peaks, indicating a high degree of graphitization (Figure 2b) [19, 20]. Notably, for CDs‐1 and CDs‐3, the (002) reflection associated with interlayer stacking is relatively sharp, while the (100) reflection related to in‐plane ordering is broadened. Such anisotropic peak broadening is commonly observed in carbon‐based nanomaterials and suggests better coherence along the stacking direction perpendicular to the graphene layers, whereas the in‐plane crystallite size is limited and/or contains more defects, resulting in a smaller lateral coherent domain. The higher graphitization degree of CDs‐1 and CDs‐3 may originate from their surface chemistry, which promotes the formation and stacking of more ordered aromatic structures during the carbonization process. Raman spectroscopy was performed to further characterize the carbon core structure of CDs (Figure S2). All three samples show two characteristic peaks of carbon materials: the D‐band at 1337 cm^−1^ (sp^3^ C) and the G‐band at 1578 cm^−1^ (sp^2^ C), confirming the typical carbon‐core framework of CDs. The calculated I_D_/I_G_ follows the order CDs‐3 (0.22) < CDs‐1 (0.25) < CDs‐2 (0.34), which is consistent with XRD results. The FT‐IR spectra of three CDs reveals characteristic absorption peaks of O─H/N─H (∼3400 cm^−1^), C═O (1701 cm^−1^), C═N (1642 cm^−1^), C─N (1270 cm^−1^), and C─O (1105 cm^−1^) (Figure 2c) [21]. Notably, CDs‐1 exhibits a strong and sharp C═O stretching vibration peak at 1693 cm^−1^, indicating significant enrichment of aldehyde groups on the surface of this sample. As the proportion of amino groups in the precursor increases, this peak gradually weakens in CDs‐2 and CDs‐3, and almost disappears in CDs‐3. Meanwhile, CDs‐3 shows a clear double peak of N─H stretching vibration in the range of 3300–3500 cm^−1^, and the C─N vibration peak significantly increases, collectively confirming that its surface is dominated by amino functional groups.

The XPS survey spectra (Figure 2d) confirm that all CDs are primarily composed of C, N, and O, showing characteristic C 1s, N 1s, and O 1s peaks at 284.7, 399.2, and 531.9 eV, respectively [22, 23]. Elemental quantitative analysis shows that as the proportion of 2,3‐diaminonaphthalene in the precursor increases, from CDs‐1 to CDs‐3, the N content gradually increases from 5.3% to 11.0%, while the O content decreases correspondingly from 9.4% to 4.4% (Table S1). The C content remains stable among the three, with C 1s proportions of 85.3%, 85.7%, and 84.6% for CDs‐1, CDs‐2, and CDs‐3, respectively. High‐resolution C 1s spectra (Figure S3) can be deconvoluted into three components assigned to C═C/C─C (284.7 eV), C─O/C─N (286.4 eV), and C═O (288.8 eV). The N 1s spectra indicate that N exists predominantly as C─N─H_2_ (398.1 eV), C_2_─N─H (399.6 eV), and C═N─C (400.6 eV) (Figure 2e) [24]. The O 1s spectra can be fitted with contributions from C═O (532.2 eV) and C─O (533.7 eV) (Figure 2f). Crucially, the fraction of the C═O component in O 1s is highest for CDs‐1 and decreases markedly for CDs‐3; correspondingly, the N 1s spectrum of CDs‐3 dominates the component representing free amine/amide, while CDs‐1 is dominated by pyrrole‐N doped in the carbon core. This result demonstrates that an aldehyde‐rich reaction environment induces the formation of an electron‐rich acceptor (aldehyde) surface, while an excess of amino groups leads to the formation of an electron‐rich donor (amine) surface [25].

In the ^1^H NMR spectrum, the aldehyde proton (─CHO) signal at a chemical shift of approximately 10 ppm can be observed in CDs‐1, indicating the presence of unreacted aldehyde groups. In contrast, no significant aldehyde signals were detected in CDs‐2 and CDs‐3, suggesting that the surface functional groups are in a relatively balanced state (Figure 2g; Figure S4). It is worth noting that all CDs exhibit amine proton signals in the 4.7–5 ppm range, which may be attributed to the instability of the Schiff base (─C═N─) formed during the synthesis of CDs, leading to the decomposition of some imine bonds and the release of some amino groups. This dynamic balance of surface functional groups implies that the surface chemistry of the CDs is not entirely static. However, within our experimental systems (ethanol solutions and the solid state), such dynamic changes are limited and occur on a slow timescale. For CDs‐1, the aldehyde‐rich surface acts as a strong hydrogen‐bond acceptor, and the hydrogen‐bonding network formed with the dynamically released amino groups possesses thermodynamic stability far exceeding the disturbance caused by any reversible processes. Consequently, the resulting ordered, hydrogen‐bond‐directed assembly remains dominant and stable. For CDs‐3, whose surface is already enriched with amino groups, the release of a small number of additional amino groups does not significantly alter its surface electron density or the driving force for *π*–*π* stacking. Therefore, under the experimental timescales and aggregation conditions examined in this work, such dynamic surface changes do not induce a macroscopic, reversible transition between the distinct assembly modes characterized in this study.

The chemical evolution pathway of CDs was elucidated through time‐dependent NMR spectroscopy conducted over a 0–6 h reaction period, with CDs‐2 selected as a representative model for in‐depth analysis due to its intermediate stoichiometry, which best elucidates the common structural evolution pathway applicable to all CDs (Figures S5–S9; Figure 2h) [26]. The process was initiated by a rapid intramolecular Mannich reaction between 2,3‐diaminonaphthalene and *o*‐phthalaldehyde, leading to the formation of a macrocyclic Schiff base intermediate within the first 10 min. Subsequently, this macrocyclic structure was subjected to Borch reduction in the alcoholic solvent, triggering a ring‐opening process that yielded key acetal and hemiacetal structural units. Clear evidence for this transformation was provided by characteristic NMR signals (^1^H: 7.9–8.0 and 5.8 ppm; ^13^C: 102.5 and 101.4 ppm; see Supporting Information). Finally, under the reaction conditions, intermolecular cross‐linking was facilitated by the thermolabile acetal motifs, resulting in the formation of a carbon dot core structure with a size of approximately 2.5 nm. Time‐dependent optical spectra complemented these structural insights: UV–vis absorption (Figure S5c) remained largely unchanged across 1–6 h, except for a slightly enhanced peak at ∼350 nm at 0.5 h—attributed to the transient presence of small macrocyclic intermediates in the early stage. In contrast, fluorescence emission (Figure S5d) showed a clear red shift, consistent with the progressive expansion of the conjugated system during the final intermolecular cross‐linking step. This red shift correlated with the formation of a compact, fully conjugated carbon dot core. Although an identical core structural unit was shared by the three types of CDs, significant differences in surface chemistry were exhibited due to variations in precursor ratios. The surface of CDs‐1 was characterized by abundant benzaldehyde groups with relatively fewer aniline‐like moieties, while the surfaces of CDs‐2 and CDs‐3 were predominantly composed of aniline‐derived structures, consistent with the higher precursor amine ratio. Among these, a lower density of surface amino groups was contained in CDs‐2, whereas a higher abundance was exhibited by CDs‐3. The differences in surface chemical composition govern the subsequent aggregation pathways and ultimately the optical properties.

To rigorously eliminate this possibility, we conducted controlled experiments by synthesizing small‐molecule analogues using identical precursors under conditions favoring monomeric product formation (denoted as DP‐1, DP‐2, and DP‐3, corresponding to the ─NH_2_: ─CHO stoichiometries of 3:7, 5:5, and 7:3, respectively). As shown in Figure S10, distinct differences were observed in both solid‐state and solution‐state optical behavior. Under UV illumination, the SSF of the small‐molecule controls exhibited markedly different colors and intensities compared to their CD counterparts. In dilute ethanol solutions, the fluorescence emission spectra of the controls displayed significant blue shifts (Δλ = 20–40 nm) relative to the CDs, with no spectral overlap in either absorption maxima or emission peaks. These findings conclusively rule out the contribution of residual small‐molecule impurities to the CDs' photoluminescence. The polymerization process was further quantitatively tracked by gel permeation chromatography (GPC) (Figure S5b). At 0.5 h, the molecular weight (Mw) was 310 Da, corresponding to early‐stage oligomers. The Mw increased to 400 Da at 1 h, then to 600 Da at 2 h, reflecting chain growth through dehydration condensation. Subsequently, the Mw decreased to 520 Da at 4 h and 500 Da at 6 h, attributed to crosslinking‐induced structural tightening and formation of a compact conjugated network. This trend quantitatively supports the proposed structural evolution pathway.

### 2.2 Solution‐State Photophysical Properties

**FIGURE 3:**
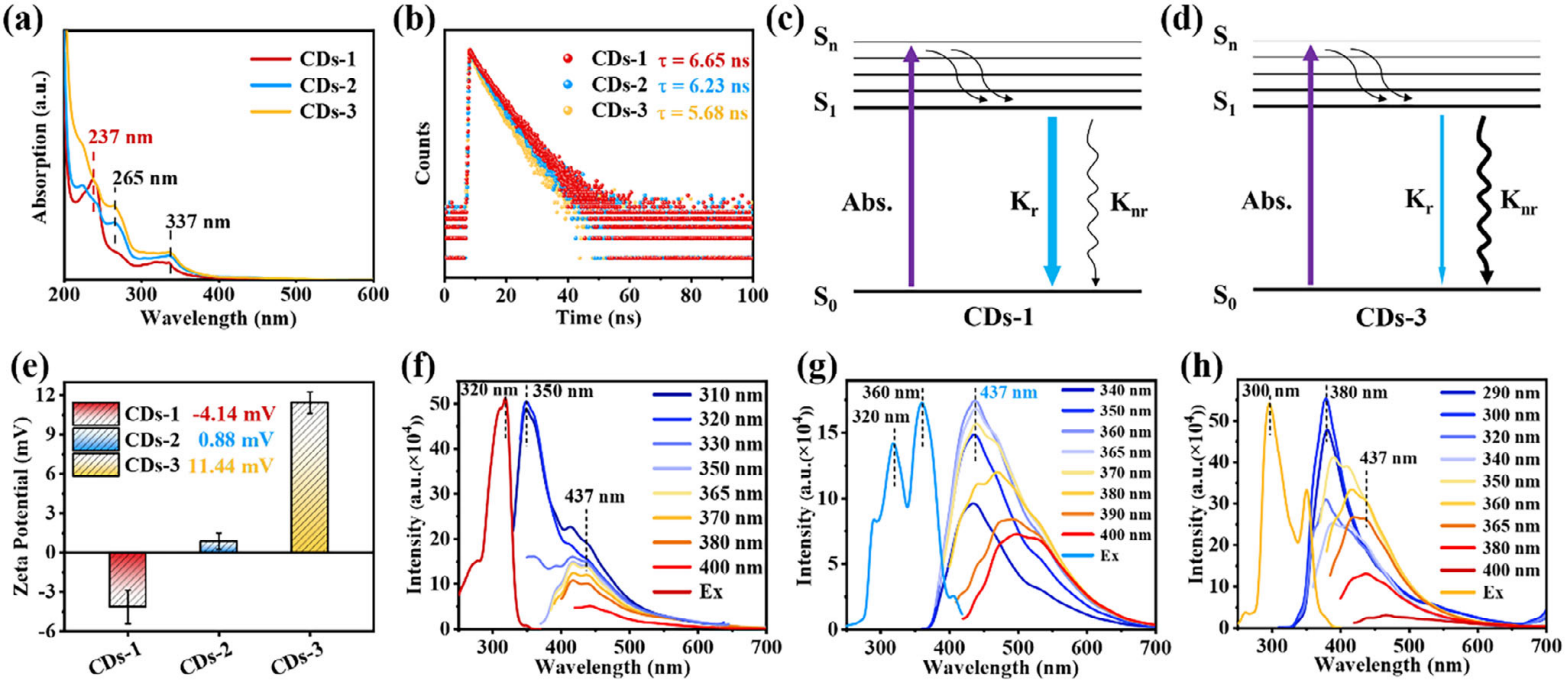
(a) UV−vis absorption spectra and (b) time‐resolved fluorescence spectra of CDs‐1, CDs‐2, and CDs‐3 in ethanol (*c* = 0.1 mg·mL^−1^). Schematic of excited‐state decay competition: radiative (k_r_) vs. non‐radiative (k_n__r_) pathways in (c) CDs‐1 and (d) CDs‐3. (e) Zeta potential of CDs‐1, CDs‐2, and CDs‐3 in ethanol (*c* = 0.1 mg·mL^−1^). PL emission spectra of (f) CDs‐1, (g) CDs‐2 and (h) CDs‐3 in ethanol (*c* = 0.1 mg·mL^−1^).

The UV–vis absorption spectra of the three CDs exhibited similar absorption peaks at 237, 265, and 337 nm, attributed to the *π*–*π* ^*^ transition of the aromatic carbon core and the n–*π* ^*^ transition of C═O/C═N bonds, indicating their common basic conjugated structure (Figure 3a) [27, 28, 29]. However, the unique surface chemical properties determined by the precursor ratio primarily influenced their subsequent excited‐state dynamics and colloidal properties. CDs‐1 (rich in aldehyde groups) exhibited the longest lifetime (6.65 ns), while CDs‐3 (rich in amino groups) had the shortest lifetime (5.68 ns), and CDs‐2 (amino/aldehyde groups = 1:1) had a lifetime (6.23 ns) between the two (Figure 3b). The fluorescence lifetime (τ) is a comprehensive reflection of the excited‐state deactivation process, governed by the radiative decay rate (K_r_) and the non‐radiative decay rate (K_nr_) according to the relationship: τ = 1/(K_r_ + K_nr_). Therefore, the observed decrease in lifetime directly indicates an increase in the total decay rate (K_r_ + K_nr_). Given that the three CDs possess similar emission wavelengths and absorption cross‐sections, their k_r_ values can be reasonably assumed to be comparable. Consequently, the shortest lifetime of CDs‐3 is primarily attributed to its significantly enhanced non‐radiative decay rate (K_nr_). This trend suggests that the efficiency of non‐radiative decay channels increases with the increase in the proportion of surface amine groups, primarily due to the photoinduced electron transfer process from electron‐rich surface amine groups to the carbon core, which competes with radiative recombination and ultimately leads to fluorescence quenching (Figure 3c,d). Additionally, zeta potential measurements directly confirmed the gradual changes in surface chemical properties: CDs‐1 exhibited a negative charge (−4.14 mV), consistent with its surface enrichment in electron‐withdrawing aldehyde groups; CDs‐2 had a surface charge close to neutral (+0.88 mV), reflecting a balanced surface composition; whereas CDs‐3 showed a pronounced positive charge (+11.44 mV), confirming the dominance of protonated amino groups (Figure 3e) [30]. This continuous change in surface polarity from negative to positive not only determines their different aggregation behaviors but may also affect the direction and efficiency of intramolecular or intermolecular charge transfer.

The fluorescence emission and 3D fluorescence spectra of three types of CDs indicate that their luminescence originates from different fluorescence centers, which are regulated by their surface chemical properties [31, 32]. Although all three types of CDs exhibit excitation wavelength‐dependent emission, and their emission peaks are all at 437 nm under excitation at 365 nm, indicating that they share a common conjugated core structure, they differ significantly in their intrinsic excitation maxima and the number of fluorescence centers. CDs‐1, rich in aldehyde groups, show a single excitation maximum at 320 nm and only one major fluorescence center, indicating a uniform surface state conducive to the formation of a clear radiative recombination pathway (Figure 3f; Figure S11a). In contrast, CDs‐3, with an amine‐rich surface, exhibits an excitation maximum at 300 nm and two fluorescence centers, indicating a more complex electronic environment where competitive processes such as photoinduced electron transfer may quench certain decay channels (Figure 3h; Figure S11c). Notably, CDs‐2, with a balanced ratio of amine to aldehyde groups, shows dual excitation peaks (320 and 360 nm) and two fluorescence centers, indicating that both surface functional groups contribute to the dynamics of the excited state (Figure 3g; Figure S11b). These observations strongly demonstrate that by adjusting the stoichiometric ratio of precursors, we can tailor the number and properties of fluorescence centers in CDs, thereby precisely controlling their excitation characteristics and ultimately guiding their optical functions to serve specific applications.

### 2.3 Aggregation Pathways From Solution to Solid State

**FIGURE 4:**
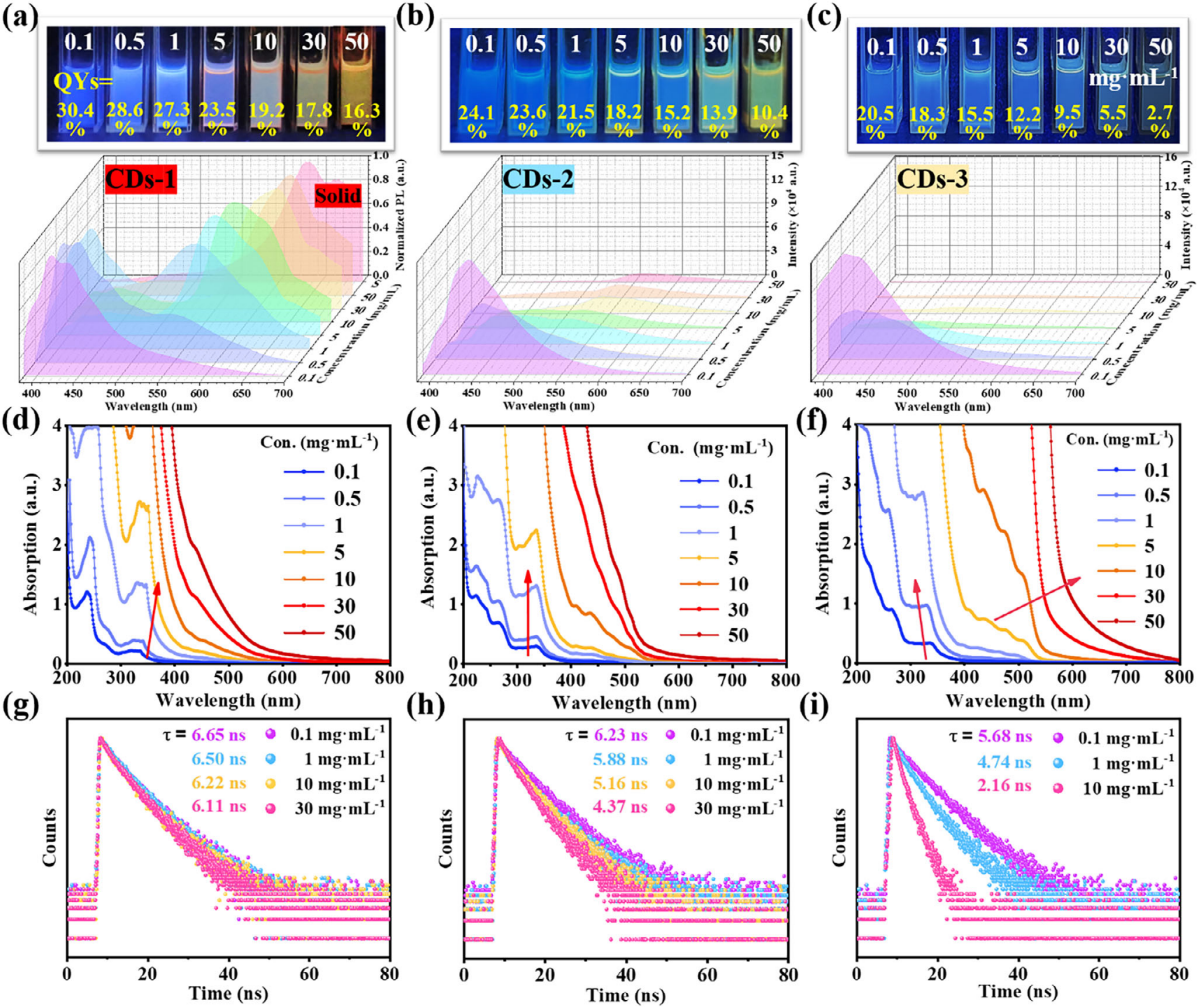
Photographs and fluorescence spectra (λ_ex_ = 365 nm) of different concentrations of ethanol solutions of (a) CDs‐1, (b) CDs‐2, and (c) CDs‐3 under 365 nm UV lamp irradiation. UV–vis absorption spectra of (d) CDs‐1, (e) CDs‐2, and (f) CDs‐3 ethanol solutions of different concentrations. Note: At higher concentrations, partial absorption signals may exceed the linear detection range of the spectrophotometer. Time‐resolved fluorescence spectra of (g) CDs‐1, (h) CDs‐2, and (i) CDs‐3 ethanol solutions of different concentrations.

To investigate the evolution of the optical properties of CDs from dilute solution to the solid state, we conducted a concentration‐dependent study (Figure 4) [33, 34]. In the lower concentration range (0.1–5.0 mg·mL^−1^), the absorption spectra of the three CDs exhibited distinct concentration‐dependent behaviors, reflecting their different aggregation pathways. For CDs‐1, a progressive redshift was observed with increasing concentration (Figure 4d). This redshift is characteristic of a staggered molecular arrangement, where exciton coupling creates lower‐energy transition pathways, thereby red‐shifting the absorption spectrum [14]. In contrast, within the same concentration range (0.1–5.0 mg·mL^−1^, Figure 4f), CDs‐3 exhibited a progressive blueshift. This blueshift is a hallmark of face‐to‐face stacking, where exciton coupling favors higher‐energy transitions [14]. The gradual nature of this blueshift suggests that the formation of such close‐packed aggregates is not an abrupt process but rather a progressive ordering of particle packing as concentration increases. CDs‐2, with balanced surface functionality, exhibited intermediate behavior in this concentration range with no clear redshift or blueshift (Figure 4e). As the concentration further increased into the higher range (5.0–50.0 mg·mL^−1^), CDs‐1 and CDs‐2 displayed only a moderate redshift to approximately 450 nm. In stark contrast, the absorption of CDs‐3 extended remarkably into the visible region, reaching up to 700 nm. This long‐wavelength redshift is a consequence of efficient ICT within the compact aggregates formed by strong *π*–*π* stacking, where the electron‐rich amine surfaces establish a continuous donor–acceptor interface with the electron‐deficient aromatic cores of adjacent particles, effectively narrowing the optical bandgap. In comparison, CDs‐2, with its balanced surface chemistry, fails to form the extended, ordered hydrogen bond network seen in CDs‐1. Instead, its aggregation is driven by non‐directional van der Waals forces and weaker hydrogen bonds, which are insufficient to overcome *π*–*π* stacking. As a result, CDs‐2 maintains strong UV‐specific absorption at lower concentrations, highlighting its potential as a UV‐shielding material.

Photoluminescence (PL) spectra under the concentration gradient further underscored the divergence in aggregation (Figure 4a–c). CDs‐1 exhibited remarkable concentration‐dependent emission, with its maximum emission wavelength redshifting from 418 nm (blue) in dilute solution to 628 nm (red) at high concentration, while the photoluminescence quantum yield (PLQY) gradually decreased, finally stabilizing at 635 nm in the solid state (PLQY = 15.2%). This continuous redshift across the entire visible spectrum is attributed to the formation of ordered, hydrogen‐bond‐directed assemblies, which arise from the head‐to‐tail molecular packing that creates new, lower‐energy exciton transition pathways. This specific packing is stabilized and enhanced by the rigid hydrogen‐bond network of its aldehyde‐rich surface, effectively outcompeting non‐radiative decay pathways. Conversely, both CDs‐2 and CDs‐3 exhibited a rapid decrease in PLQY and eventual solid‐state quenching. This behavior resulted from the formation of compact aggregates, enabled by unchecked *π*–*π* stacking due to the lack of competing intermolecular forces on their surfaces. The resulting strong exciton coupling in these aggregates provides a highly efficient non‐radiative pathway, leading to complete fluorescence quenching. Time‐resolved fluorescence measurements across concentration gradients reveal distinct excited‐state dynamics (Table 1 and Figure 4g–i) [35, 36, 37]. CDs‐1 maintains a stable lifetime (∼6.65 to 6.11 ns) (Figure 4g), indicating a rigid, hydrogen‐bonded network that suppresses non‐radiative decay. In contrast, CDs‐3 exhibits a drastic lifetime decrease (5.68 to 2.16 ns) due to efficient non‐radiative pathways from its face‐to‐face stacked assembly (Figure 4i). CDs‐2 shows an intermediate, decreasing trend, reflecting a competitive balance between radiative and non‐radiative processes consistent with its balanced surface chemistry (Figure 4h). Biexponential fitting analysis confirmed that for CDs‐3, which is dominated by compact *π*–*π* stacking, the contribution of the short‐lived component (∼1.25–2.99 ns) increased significantly with concentration, while both the short‐ and long‐lived components themselves shortened considerably. This is attributed to the greatly enhanced ICT process facilitated by the continuous donor–acceptor interface formed upon aggregation, thereby providing an efficient non‐radiative dissipation pathway for the excited state [38]. In contrast, CDs‐1, which form ordered assemblies via directional hydrogen bonding, suppressed close *π*–*π* contact and the formation of strong ICT, preserving its long‐lived component (∼6.94–6.97 ns) and thus effectively inhibiting ACQ.

**TABLE 1:** Multi‐exponential decay curves of CDs solution at different concentrations.

	Con.(mg·mL^−1^)	τ_1_/ns	B_1_/%	τ_2_/ns	B_2_/%	τ_av_/ns
CDs‐1	0.1	1.87	5.65	6.94	94.35	6.65
	1	3.39	13.07	6.97	86.93	6.50
	10	2.80	17.79	6.96	82.21	6.22
	30	3.22	22.97	6.97	77.03	6.11
CDs‐2	0.1	2.32	21.37	7.92	78.63	6.23
	1	3.24	40.63	7.68	59.37	5.88
	10	2.83	52.30	7.71	47.70	5.16
	30	2.48	60.15	7.22	39.85	4.37
CDs‐3	0.1	2.99	32.22	6.96	67.78	5.68
	1	2.80	41.31	6.11	58.69	4.74
	10	1.25	40.25	2.78	59.75	2.16

Herein, τ_i_ represents the corresponding lifetime of different components, B_i_ stands for the corresponding pre‐exponential factor, τ_av_ denotes the average lifetime.

**FIGURE 5:**
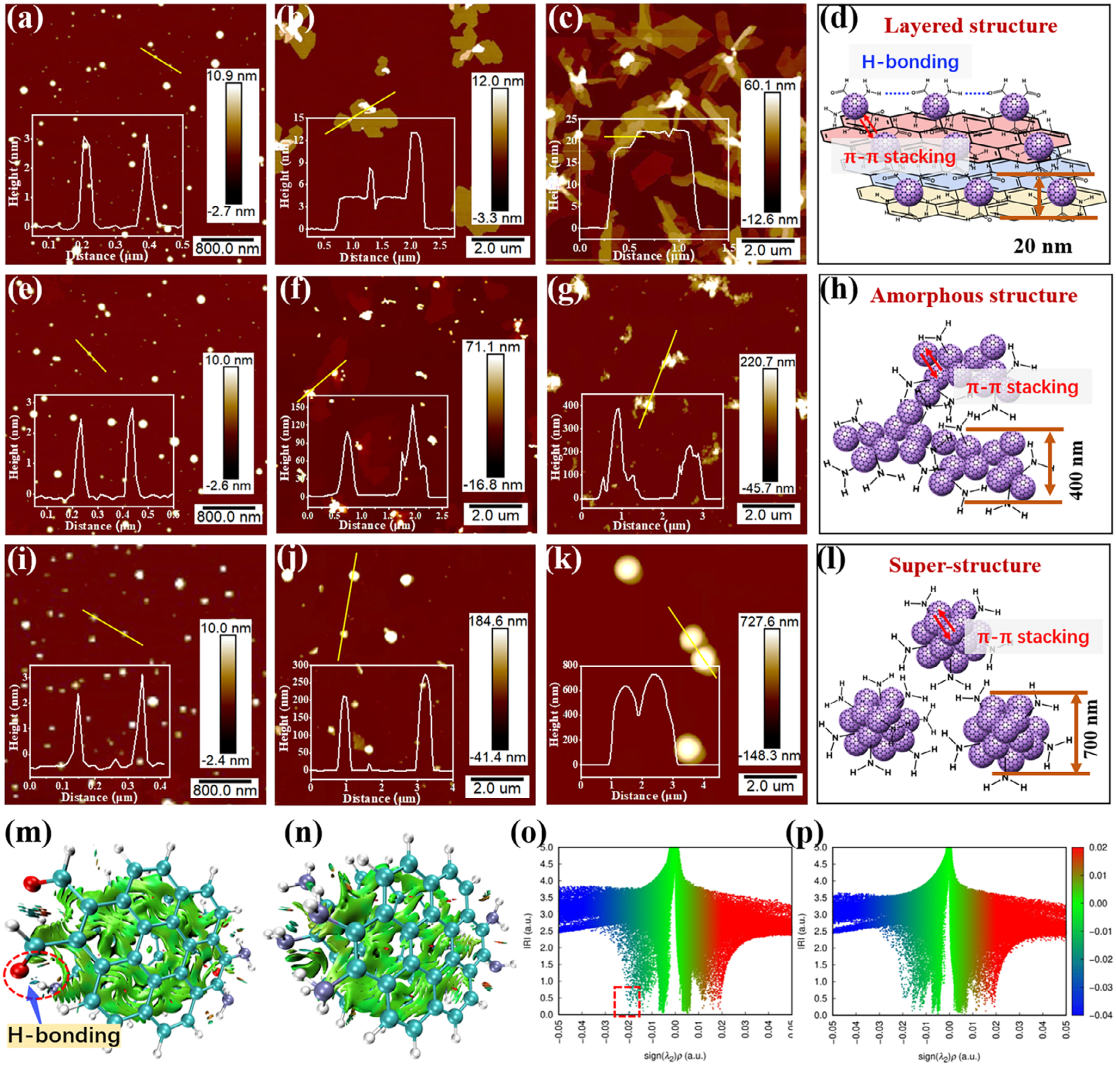
AFM images of (a–c) CDs‐1, (e–g) CDs‐2, and (i–k) CDs‐3 deposited from ethanol solutions (0.1, 1, and 10 mg·mL^−1^) onto silicon substrates. Schematic diagram of the aggregation state of (d) CDs‐1, (h) CDs‐2, and (l) CDs‐3. NCI isosurface and scatter plots of (m, o) CDs‐1 and (n, p) CDs‐3 aggregates, colored by the sign of the second derivative of the electron density (sin(λ_2_)ρ) to distinguish weak interactions. Blue indicates strong attractive interactions (hydrogen bonds), green represents weaker van der Waals interactions, and red corresponds to strong repulsive interactions (spatial effects).

Atomic force microscopy (AFM) was employed to visualize the aggregation morphologies of the three types of CDs. As shown in Figure 5a–d, CDs‐1 assemble into highly uniform, sheet‐like layered structures with a thickness of approximately 20 nm. This anisotropic two‐dimensional morphology is characteristic of oriented, head‐to‐tail molecular packing [39]. Importantly, this morphological feature aligns with the spectroscopic signatures observed for CDs‐1: concentration‐dependent UV–vis absorption shows progressive redshift without clear isosbestic points (Figure 4d), and the fluorescence lifetime remains stable upon aggregation, both of which are hallmarks of exciton coupling in staggered (head‐to‐tail) packing systems. Furthermore, concentration‐dependent ^1^H NMR confirms the formation of directional N─H⋯O═C hydrogen bonds during aggregation (Figure S11), which provide the driving force for this ordered assembly. Collectively, the morphological, spectroscopic, and intermolecular interaction evidence consistently support that CDs‐1 undergo ordered assembly driven by directional hydrogen bonding.

In contrast, CDs‐3 form compact, isotropic spherical aggregates with heights up to approximately 700 nm (Figure 5i–l). This morphology is consistent with compact assembly driven by enhanced face‐to‐face *π*–*π* stacking [39], as further supported by the concentration‐dependent absorption blueshift at low concentrations (Figure 4f) and the dramatic fluorescence lifetime shortening upon aggregation, indicative of efficient ICT within these tightly packed aggregates. CDs‐2, with balanced surface functionality, assemble into amorphous aggregates of approximately 400 nm in height (Figure 5e–h), reflecting a disordered aggregation process where neither hydrogen bond directed ordering nor strong *π*–*π* stacking dominates.

To investigate the intermolecular interactions driving CDs aggregation, we conducted concentration‐dependent ^1^H NMR experiments. For CDs‐1, their NMR spectra in deuterated methanol showed multiple independently distinguishable signals in the aromatic region (6–8 ppm), accompanied by a sharp characteristic peak attributed to ─NH_2_ protons at 4.78–5.23 ppm. Importantly, as the concentration increased, the ─NH_2_ proton signal at 5.21 ppm continuously shifted toward higher fields (Figure S12). This change in chemical shift indicates the formation of hydrogen bonds between molecules during the aggregation process. These directional hydrogen‐bonding interactions drive the ordered assembly of CDs‐1, providing the key driving force for the growth of two‐dimensional layered films and underpinning the distinctive solid‐state red emission observed. In contrast, under the same conditions, the ^1^H NMR chemical shifts of CDs‐2 and CDs‐3 did not change with increasing concentration (Figures S13 and S14). The absence of chemical shifts confirms that intermolecular hydrogen bonds did not participate in the aggregation process of these two CDs.

### 2.4 Elucidating the Mechanism: From Surface Chemistry to Aggregation‐Dependent Properties

To elucidate the mechanism underlying the distinct aggregation behaviors, we employed density functional theory (DFT) calculations to model the intermolecular interactions. Two representative dimer models were constructed: one for CDs‐1, featuring both amino and aldehyde groups on a conjugated core, and another for CDs‐3, bearing only amino groups. Non‐covalent interaction (NCI) analysis based on the reduced density gradient (RDG) revealed significant differences. For CDs‐1, distinct blue isosurfaces between aldehyde and amino groups confirmed the presence of directional hydrogen bonding (Figure 5m,o). In stark contrast, such hydrogen‐bonding signatures were nearly absent in CDs‐3, where interactions were dominated by green regions indicative of non‐directional van der Waals forces and *π*–*π* stacking (Figure 5n,p).

Based on systematic structural characterization, photophysical properties in solution, concentration‐dependent experiments, and theoretical calculations, this study clearly reveals the intrinsic relationship between the aggregation behavior and optical function of three types of CDs. Due to its aldehyde‐rich surface, CDs‐1 primarily self‐assemble through directional hydrogen bonding, forming layered structures characterized by staggered molecular packing. This ordered stacking structure effectively isolates the luminescent groups spatially, significantly suppressing non‐radiative *π*–*π* stacking, thereby achieving efficient and red‐shifted SSF emission through radiative low‐energy exciton transition pathways. Although the residual *π*–*π* interactions during aggregation lead to a partial decrease in fluorescence quantum yield, the emission wavelength can cover the entire visible light region with changes in concentration. This unique property endows CDs‐1 with clear application potential in constructing multicolor LEDs.

In contrast, CDs‐3 leveraging the strong electron‐donating properties provided by their surface‐enriched amino groups, significantly enhance the *π*–*π* stacking driving force between carbon cores, leading to the formation of compact aggregates with face‐to‐face packing. Such closely packed superstructures provide efficient migration pathways for excitons and promote ICT, thereby channeling excited‐state energy into non‐radiative decay pathways. This mechanism not only results in complete fluorescence quenching but also significantly broadens the absorption range to the visible‐near‐infrared region, making them highly suitable for photothermal conversion applications.

On the other hand, CDs‐2 exhibits behavior that falls between the two. Due to the balanced distribution of surface functional groups and the absence of a clearly dominant interaction, the competition between hydrogen bonding and *π*–*π* stacking leads to the formation of amorphous aggregates. In this structure, neither radiative recombination nor non‐radiative decay pathways dominate. Macroscopically, this manifests as the coexistence of strong ultraviolet absorption and partial fluorescence quenching, which precisely meets the optical requirements for efficient ultraviolet shielding materials.

This programmable aggregation, dictated by precursor stoichiometry, demonstrates a general design principle. The versatility of *o*‐phthalaldehyde, which provides essential hydrogen‐bonding sites, as a precursor for generating SSF CDs was further confirmed through its successful co‐carbonization with various common carbon sources, including *o*‐phenylenediamine, urea, lignin, and chitosan [40, 41, 42]. While these carbon sources alone failed to exhibit SSF upon carbonization, their co‐carbonization with *o*‐phthalaldehyde consistently produced CDs with notable solid‐state emission (Figure S15). A controlled experiment demonstrated that the carbonization of *o*‐phthalaldehyde alone resulted in only a viscous and non‐dried liquid, whose concentrated state exhibited a faint cyan fluorescence and was unable to form a solid powder with strong emission and stability. This confirms that *o*‐phthalaldehyde alone is insufficient to form ordered hydrogen‐bond‐stabilized assemblies. Therefore, *o*‐phthalaldehyde provides a crucial aldehyde (─CHO) functional group during the co‐carbonization process. These aldehyde groups are bound to the surface of the growing CDs and act as the necessary “hooks” for constructing the directional hydrogen‐bond network, while the co‐carbonization partner (such as compounds rich in amines) mainly contributes to the formation of the carbon core and introduces complementary amine (─NH_2_) groups. The control experiment, where the aldehyde group was replaced with other hydrogen‐bonding groups such as hydroxyl or carboxyl, failed to achieve red SSF of comparable intensity, highlighting the unique role of the aldehyde group in guiding ordered, hydrogen‐bond‐directed assembly (Figure S16). The key lies in the fact that the aldehyde group not only provides strongly directional hydrogen‐bonding sites but also enables in situ formation of covalent imine bonds (C═N─) with amino groups during synthesis. This precisely locks the “head‐to‐tail” orientation between carbon dot units, which is an indispensable mechanism for constructing efficient staggered‐packing structures and achieving red‐shifted emission.

For achieving extended absorption into the long‐wavelength region, however, amine‐enriched surfaces are required. To verify this, we first compared the concentration‐dependent spectral shifts of naphthalene and 2,3‐diaminonaphthalene (Figure S17). While naphthalene showed a negligible redshift, 2,3‐diaminonaphthalene exhibited a substantial bathochromic shift extending to approximately 500 nm. Similarly, *o*‐phthalaldehyde alone displayed minimal concentration‐dependent spectral shifts. Although the spectral changes of small molecules in concentrated solutions primarily arise from intermolecular excitonic coupling, while the redshift of CDs during aggregation stems from close nanoparticle packing and the resulting strong ICT at the nanoscale—implying different underlying mechanisms—this comparison nonetheless reveals a common physicochemical factor: the electron‐donating nature of amine groups. In 2,3‐diaminonaphthalene, the amine groups enhance the electron density of the aromatic ring, thereby strengthening intermolecular *π*–*π* interactions. Analogously, in the CDs system, the abundant amine groups on the surface of CDs‐3 similarly function to increase the electron density of the carbon core, which in turn reinforces the *π*–*π* stacking driving force between CD particles and promotes the formation of compact aggregates with strong ICT interfaces. We further compared the CDs derived separately from 2,3‐diaminonaphthalene (CDs‐4) and *o*‐phthalaldehyde (CDs‐5) under identical carbonization conditions (Figure S18). Notably, CDs‐4 exhibited a far more pronounced concentration‐dependent redshift than CDs‐5, reinforcing that amine‐enriched surfaces are crucial for extending absorption into longer wavelengths. Based on the above insights, we further conducted a co‐carbonization process using 2,3‐diaminonaphthalene with several representative biomass‐derived carbon sources (including citric acid, glucose, and gallic acid), successfully preparing CDs‐6, CDs‐7, and CDs‐8 (Figure S19). As expected, all three types of CDs exhibited significant concentration‐dependent red shifts in their absorption spectra. Together, these control experiments validate the general applicability of our precursor‐guided approach to tailor CDs aggregation and optical properties for targeted functions.

### 2.5 Applications of Multifunctional CDs for LEDs, Photothermal Conversion, and UV Shielding

**FIGURE 6:**
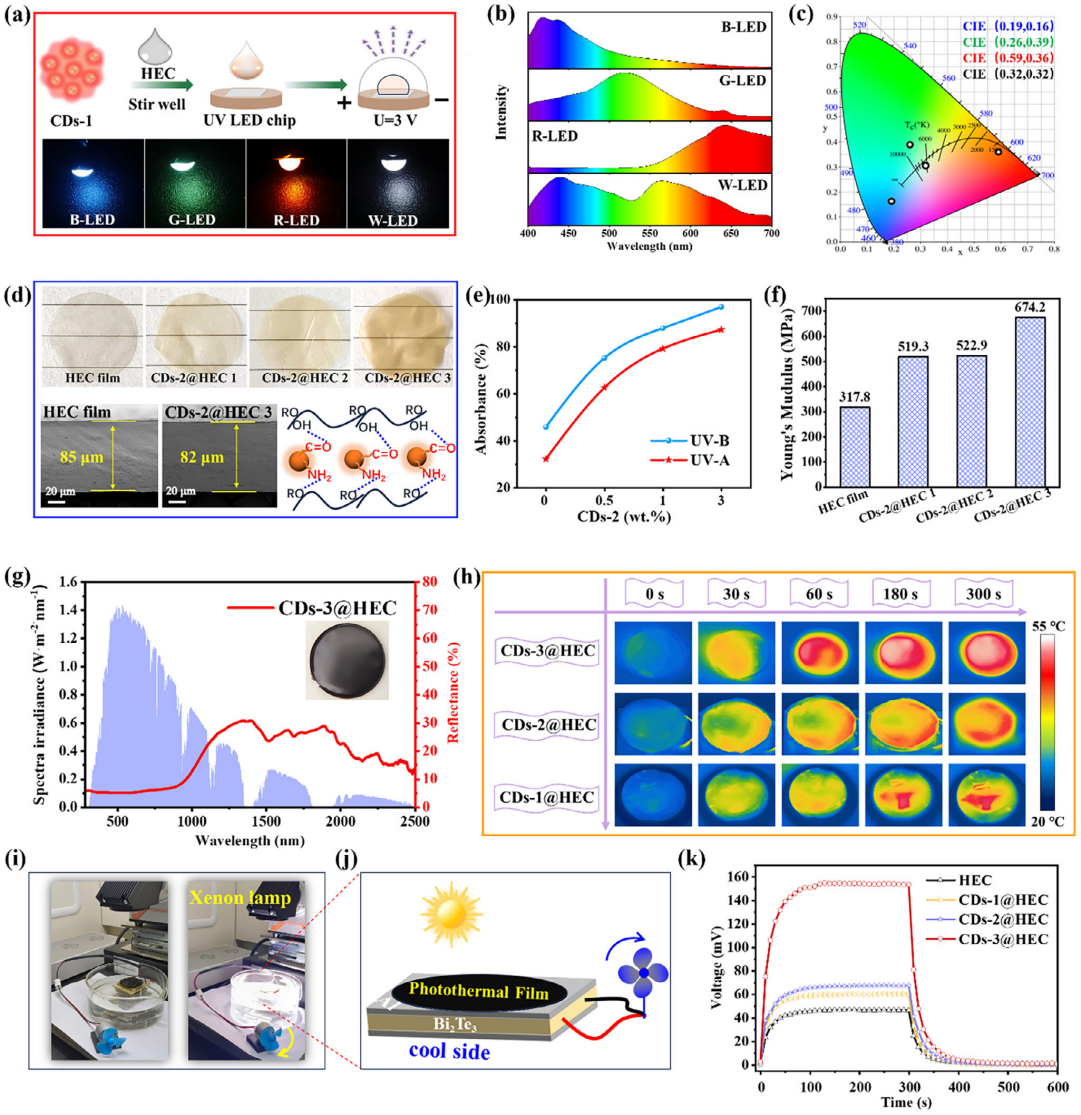
(a) Schematic diagram of the preparation method of LEDs and photographs of different color LEDs. (b) Corresponding emission spectra of LED lights. (c) LED color coordinates. (d) Pristine cellulose film, 0.5, 1, and 3 wt.% CDs‐2 loaded cellulose films, their cross‐sectional scanning electron microscope (SEM) images, and a schematic diagram of hydrogen bonds formed between CDs‐2 and HEC. (e) UV‐A and UV‐B blocking activity of different films. (f) The Young's modulus of the CDs‐2@HEC films. (g) UV–vis–NIR diffuse reflectance spectrum of CDs‐3@HEC film, with the yellow curve representing the solar spectrum. (h) Infrared thermal imaging photos of the CDs‐1@HEC, CDs‐2@HEC, and CDs‐3@HEC films under simulated sunlight with an intensity of 100 mW·cm^−2^. (i) Thermal‐electric conversion device. (j) Schematic diagram of the principle of the photo‐thermoelectric conversion device; (k) 100 mW·cm^−2^ solar radiation 300 s and then turn off the light source, open circuit voltage change curve.

Guided by the distinct aggregation‐dependent properties, we first verified the excellent structural and fluorescence stability of the CDs under both ambient and working conditions, confirming their reliability for practical solid‐state applications (Figure S20). We then integrated the three types of CDs into an environmentally friendly and renewable hydroxyethyl cellulose (HEC) matrix, fabricating a series of functional composite films for targeted applications. This approach not only demonstrates the practical utility of our CDs but also aligns with green material principles [43]. Leveraging the concentration‐dependent full‐spectrum fluorescence properties of CDs‐1, we fabricated multicolor LEDs [4, 44]. As illustrated in Figure 6a, ethanol solutions of CDs at different concentrations were mixed with an aqueous solution of HEC (5 wt.%) and drop‐casted onto UV‐LED chips. After drying at room temperature, LEDs emitting different colors were obtained: blue LED (B‐LED) at low concentration, green LED (G‐LED) at medium concentration, and red LED (R‐LED) at high concentration. A white LED (W‐LED) was also prepared by coating a high‐concentration CDs‐1 solution onto a blue LED chip. Notably, the hydroxyethyl cellulose matrix physically isolates the CDs‐1 and partially restricts their aggregation, thereby preserving the solution‐like broadband emission essential for white‐light generation. Photographs of the four types of LEDs operating at 3 V are shown in Figure 6a. The emission spectra of the LEDs are presented in Figure 6b. The B‐LED, G‐LED, and R‐LED exhibit emission peaks at 420, 525, and 635 nm, corresponding to blue, green, and red light, respectively. Their CIE 1931 chromaticity coordinates are (x = 0.19, y = 0.16), (x = 0.26, y = 0.39), and (x = 0.59, y = 0.36). The photoluminescence spectrum of the W‐LED shows broad emission across the range of 400–700 nm, with CIE coordinates of (0.32, 0.32, Figure 6c).

The absorption and fluorescence spectra of CDs‐2 confirm its capability to effectively absorb UV radiation and convert it into green‐to‐orange‐red fluorescence. Leveraging this property, we fabricated composite films by incorporating CDs‐2 into a pure HEC matrix and evaluated their UV‐shielding performance. As illustrated in Figure 6d, the transparency of the films shifted to an orange‐red hue with increasing mass fraction of CDs‐2 (0, 0.5, 1, and 3 wt.%). Figure S21a shows that the film has a high degree of flexibility, and Figure S21b shows the state of CDs‐2@HEC 3 (3 wt. %) film after one year of placement, indicating that the film is stable and basically does not change over time, and other films are also stable. Both HEC and CDs‐2@HEC films exhibit dense and uniform cross‐sectional morphologies with a thickness of approximately 85 µm. The optical properties of the four films were evaluated by fluorescence spectroscopy, which also helps explain the wavelength conversion activity of the films in absorbing ultraviolet light. Figure S22 shows the fluorescence spectra of CDs‐2@HEC films at the excitation wavelength of 365 nm. The fluorescence of CDs‐2 in the cellulose matrix is similar to that in solution. When the concentration of CDs‐2 in the cellulose film increased from 0.5 to 3wt.%, the shape of the fluorescence spectrum did not change significantly, the red emission of the film is enhanced, clearly indicating that ultraviolet light can be converted to higher wavelengths of orange‐red emission. UV‐shielding efficacy was assessed via absorbance and transmittance measurements (Figure S23). The pure HEC film transmitted 78% of visible light but blocked only 32% of UV‐A and 46% of UV‐B radiation, indicating inadequate UV protection. In contrast, the incorporation of CDs‐2 significantly enhanced the UV‐blocking performance: as the loading increased from 0.5 to 3 wt.%, the UV‐A blocking rate improved from 63% to 87%, and the UV‐B blocking rate rose from 75% to 97% (Figure 6e). These results demonstrate that CDs‐2‐based biodegradable films can effectively attenuate harmful UV radiation [45, 46, 47]. Moreover, the addition of CDs‐2 improved the mechanical properties of the cellulose films. Stress–strain curves and Young's moduli for the four film types are presented in Figure 6f and Figure S24, respectively. With increasing CDs‐2 concentration, the tensile strength increased from 19.9 to 22.8 MPa, and the Young's modulus rose from 317.8 to 674.2 MPa (Table S2). This mechanical reinforcement is attributed to the formation of hydrogen bonds between the abundant functional groups (─OH, C═O) on CDs‐2 and the HEC matrix, which effectively strengthens the film structure [45].

The amino‐rich CDs (CDs‐3) film was characterized by UV–vis–NIR diffuse reflectance spectroscopy over the wavelength range of 250–2500 nm. As shown in Figure 6g, the film exhibits a reflectivity as low as 5% within the 300–800 nm region, along with a strong spectral overlap with the solar spectrum, indicating its excellent capacity for sunlight absorption and promising photothermal properties [8, 48, 49]. Infrared thermal images (Figure 6h) display the temperature evolution of the film under simulated solar irradiation (100 mW·cm^−2^). The temperature of the CDs‐3@HEC film increased rapidly within 60 s, reaching 38.5°C  after only 30 s of illumination. After 5 min, the temperature of the composite film rose to 49.7 °C, compared to only 30.6°C for the pure HEC film, and 39.0 °C and 38.2 °C for the CDs‐2@HEC and CDs‐1@HEC films, respectively (Figure S25), demonstrating the significant photothermal effect conferred by CDs‐3. By utilizing the temperature difference between the irradiated film and a cooling fin, a thermoelectric device was constructed to generate electricity; a schematic of the power generation setup is illustrated in Figures 6i,j. Under one‐sun illumination (100 mW·cm^−2^), the open‐circuit voltage of the device rapidly increased and stabilized at around 150 mV. When the light was turned off, the voltage dropped sharply to 10% of its maximum value within 70 s, indicating a fast photothermal‐electrical response (Figure 6k). At higher irradiation intensities of 200 and 300 mW·cm^−2^, the output voltage increased to 270 and 390 mV, respectively, further confirming the effectiveness of the photothermal conversion (Figure S26a). The device also exhibited good reproducibility over three on/off cycles under one‐sun conditions, maintaining consistent voltage rise and decay profiles, which suggests reliable performance for practical power generation applications (Figure S26b).

## 3 Conclusion

In summary, we have established a clear structure property relationship linking precursor stoichiometry to the macroscopic optical properties of CDs. By precisely controlling the molar ratio of 2,3‐diaminonaphthalene to *o*‐phthalaldehyde, we prepared a series of CDs with surface properties ranging from electron‐rich acceptors to electron‐rich donors. These surface characteristics govern the aggregation behavior at the nanoscale. Aldehyde‐rich surfaces form a three‐dimensional network structure through hydrogen bonding that preserves radiative pathways, while amine‐rich surfaces form compact aggregates through *π*–*π* stacking that lead to fluorescence quenching. These two aggregation morphologies direct distinct excited state relaxation pathways. One pathway involves radiative surface state charge transfer that produces efficient SSF, and the other involves non‐radiative intermolecular charge transfer that enables broadband absorption and efficient photothermal conversion. Our findings not only overcome the long‐standing challenge of solid‐state fluorescence quenching in carbon nanomaterials but also provide a universal design principle for the targeted engineering of CDs for optoelectronic devices and energy conversion applications.

## 4 Experimental Section

### 4.1 Materials

2,3‐diaminonaphthalene (97.0%), *o*‐phthalaldehyde (99.0%), hydroxyethyl cellulose (HEC, 99.0%), ethanol (99.7%), glycerol (99.7%), *o*‐phenylenediamine (99.0%), urea (99.0%), Citric acid (99.5), Chitosan (90%), lignin (Enzymatic hydrolysis lignin (EL) purchased from Pu'er, Yunnan), naphthalene (99.0%), glucose (AR), gallic acid (98%) were purchased from Shanghai Titan Technology Co., LTD. Unless otherwise stated, all raw materials are used as is and no purification is required.

### 4.2 Synthesis of CDs

CDs‐1, CDs‐2, and CDs‐3 were synthesized via a one‐pot solvothermal method using 2,3‐diaminonaphthalene and *o*‐phthalaldehyde as precursors at different molar ratios (NH_2_: CHO = 3: 7 for CDs‐1, 5:5 for CDs‐2, and 7:3 for CDs‐3) in ethanol. The reaction mixture was heated at 200°C for 6 h and then allowed to cool naturally to room temperature. The resulting crude solution was subjected to successive filtration through filter paper and a 0.22 µm membrane. The collected product was purified by column chromatography using silica gel as the stationary phase and ethanol as the eluent to obtain the final CDs products.

### 4.3 Computational Methodology

Density functional theory (DFT) calculations were carried out using Gaussian 09 at the B3LYP/6‐31G(d) level with Grimme's D3(BJ) dispersion correction to optimize the ground‐state geometries of all molecules [50]. To characterize the noncovalent interactions within the investigated systems, the reduced density gradient (RDG) was generated using Multiwfn 3.8(dev) [51, 52]. Furthermore, the calculation results were visualized with VMD 1.9.3. Notably, the noncovalent interactions in different systems were identified via the sign(λ_2_)ρ criterion [53].

### 4.4 Preparation of the LEDs

An ethanol solution of CDs‐1 (1.0 mg·mL^−1^) was blended with an aqueous solution of hydroxyethyl cellulose (HEC, 5 wt.%), applied onto the UV‐LED chip, and air‐dried naturally at room temperature for 24 h to produce a blue‐emitting LED. By increasing the concentration of CDs‐1, green (30 mg·mL^−1^) and red (50 mg·mL^−1^) LEDs could also be obtained. Furthermore, a white LED was achieved by coating a 50 mg·mL^−1^ of CDs‐1 solution onto a 420 nm blue LED chip.

### 4.5 Preparation of CDs@HEC Film

HEC aqueous solution (2 wt.%) was prepared by dissolving 1.0 g of HEC powder in 49 mL of ultrapure water, followed by stirring for 1 h at room temperature. Subsequently, 100 µL of glycerol was added as a plasticizer, and the mixture was vigorously stirred for another hour to obtain a homogeneous, transparent, and viscous solution. Different weight percentages of CDs‐2 ethanol solution were then incorporated into this solution, and the mixtures were stirred for 5 h. After centrifugation, the solutions were cast into Petri dishes and dried at room temperature for 72 h to form solid films. Films fabricated with CDs‐2 concentrations of 0, 0.5, 1, and 3 wt.% were designated as HEC film, CDs‐2@HEC 1, CDs‐2@HEC 2, and CDs‐2@HEC 3, respectively.

### 4.6 Preparation of Solar‐Thermal Conversion Device

A CDs‐3@HEC film containing 10 wt.% CDs‐3 was prepared in the same manner as the CDs‐2@HEC film. The film was black with a smooth surface. The commercial Bi_2_Te_3_ square plate is used as a thermoelectric conversion module. Two aluminium plates are sandwiched on both sides of the Bi_2_Te_3_ module to make a sandwich structure. CDs‐3@HEC composite film is attached to the top aluminium plate as the hot side under sun irradiation, and the cooling fin immersed in water is connected to the cold side as a simple photo‐thermoelectric conversion device.

## Conflicts of Interest

The authors declare no conflicts of interest.

## Supporting information




**Supporting File**: advs75737‐sup‐0001‐SuppMat.docx.

## Data Availability

The data that support the findings of this study are available from the corresponding author upon reasonable request.
