# Supplementary material for: Tailoring Multifunctional Carbon Dots via Precursor Stoichiometry: Switching Between Solid‐State Fluorescence and Broadband Absorption Through Aggregation Control
Source: Adv Sci (Weinh). 2026 May 15:e75737. Online ahead of print. doi: 10.1002/advs.75737 (PMC13336068; doi:10.1002/advs.75737)
Supplement: Supplementary file 1 — Supporting File: advs75737‐sup‐0001‐SuppMat.docx. [file ADVS-9999-e75737-s001.docx]

**Supporting Information**

**Tailoring Multifunctional Carbon Dots via Precursor Stoichiometry: Switching between Solid-State Fluorescence and Broadband Absorption through Aggregation Control**

*Gaixia Yang^1^, Hao Sun^1^*, Defa Hou^1^, Fulin Yang^1^, Yuan Zou^1^, Bei Zhou^1^,* *Lanxiang Liu^2^*, Xu Lin^1^* and* *Guanben Du^1^**

^1.^National Joint Engineering Research Center for Highly-Efficient Utilization Technology of Forestry Resources; Southwest Forestry University, 300 Bailong Road, Kunming 650224, Yunnan Province, China. [yanggaixia2022@163.com](mailto:yanggaixia2022@163.com), [houdefa001@163.com](mailto:houdefa001@163.com), [yangfulin0309@163.com](mailto:yangfulin0309@163.com), [yzou24@swfu.edu.cn](mailto:yzou24@swfu.edu.cn), [beizhou@swfu.edu.cn](mailto:beizhou@swfu.edu.cn).

^2.^Yunnan Key Laboratory of Breeding and Utilization of Resource Insects, Key Laboratory of Protection and Utilization of Insects (National Forestry and Grassland Administration), Research Center of Engineering and Technology of Characteristic Forest Resources (National Forestry and Grassland Administration), Institute of Highland Forest Science, Chinese Academy of Forestry, Kunming 650233, China.

Corresponding author: [sunhao@swfu.edu.cn](mailto:sunhao@swfu.edu.cn), [lanxiangliu@outlook.com](mailto:lanxiangliu@outlook.com), [linxu@swfu.edu.cn](mailto:linxu@swfu.edu.cn), [guanben@swfu..edu.cn](mailto:guanben@swfu..edu.cn).

1. Optimization of Synthesis Parameters

The key synthesis parameters (reaction temperature and reaction time) for CDs were optimized by tracking the solid-state fluorescence intensity of CDs-1, the sample expected to exhibit the strongest solid-state emission. We tested different temperature gradients (160°C, 180 °C, 200 °C, 220 °C) and reaction time gradients (4 h, 6 h, 8 h). We found that the solid-state fluorescence intensity of CDs-1 did not increase further when prolonging the reaction time beyond 6 h, and CDs-1 obtained at 200 °C for 6 h exhibited the maximum solid-state fluorescence intensity. Thus, this condition was selected as the standard synthesis parameter for all three CD samples (CDs-1, CDs-2, CDs-3) to guarantee the comparability of subsequent characterization and performance tests.

For precursor molar ratio, we systematically explored a series of feeding ratios of 2,3-diaminonaphthalene to *o*-phthalaldehyde, and selected three representative products with distinct surface chemistry, assembly behavior and luminescence properties (with –NH_2_:–CHO ratio of 3:7, 5:5, 7:3 for CDs-1, CDs-2, CDs-3, respectively) for systematic investigation in the main text.

2. Methods

The CDs were characterized by transmission electron microscopy (TEM) using FEI Tecani G2 F20 at 200 kV acceleration. The cross section of the films was characterized by scanning electron microscope (SEM) using German ZEISS Gemini SEM 300. Ultraviolet-visible spectra of CDs and thin films were recorded by Shimazu UV-2600 spectrometer. Fluorescence measurements were taken using the Shimadzu fluorescence spectrophotometer RF-6000 collection. Fourier transform infrared (FT-IR) spectra were obtained in transmission mode using the KBr particle technique on the Thermal Science Nicolet iS5 spectrometer (Waltham, MA, USA), and eight scans were accumulated at a resolution of 1 cm^-1^ to obtain a spectrum. X-ray photoelectron spectroscopy (XPS) was studied using a K-Alpha spectrometer with a single X-ray source Al Kα excitation (1486.6 eV). Binding energy calibration is based on C1s of 284.8eV. X-ray diffraction (XRD) patterns were recorded on a Bruker D8 Advance diffractometer using Cu Kα radiation (λ = 1.5406 Å) over a 2θ range of 10°–80° with a step size of 0.02°. Raman spectra were acquired using a Horiba LabRAM HR Evolution spectrometer with a 532 nm excitation laser, and the spectral range was 50–4000 cm⁻¹. Nuclear magnetic resonance (NMR) spectra, including ¹H NMR and ¹³C NMR, were obtained on a Bruker 500 MHz spectrometer using CD_3_OD as the solvent. Atomic force microscopy (AFM) images were recorded on a Bruker Dimension Icon system in tapping mode, and the samples were prepared by spin-coating a dilute CD solution onto a freshly cleaved mica surface. UV-vis-NIR spectra were measured on Shimadzu UV-3600i Plus.

**3. UV Blocking Performance of the Film and Evaluation**

UV−vis transmittance spectra of different types of CDs-2@HEC films were investigated by UV−vis spectrophotometry using air as a reference. Transmittance spectra were used to evaluate the UV protection afforded by the films. Blocking activities of the films were evaluated using the following equations.

UV-A blocking (315−400 nm) calculation

$$UV-A blocking \left( \% \right)=100-\frac{\int_{315}^{400} T\left( \lambda\right)d\lambda}{\int_{315}^{400} d\lambda}(\%)$$

UV-B blocking (280−315 nm) calculation

$$UV-B blocking \left( \% \right)=100-\frac{\int_{280}^{315} T\left( \lambda\right)d\lambda}{\int_{280}^{315} d\lambda}(\%)$$

where T(λ) is the average spectral transmittance of the film, dλ is the bandwidth, and λ is the wavelength.

**Supporting Figures**

**
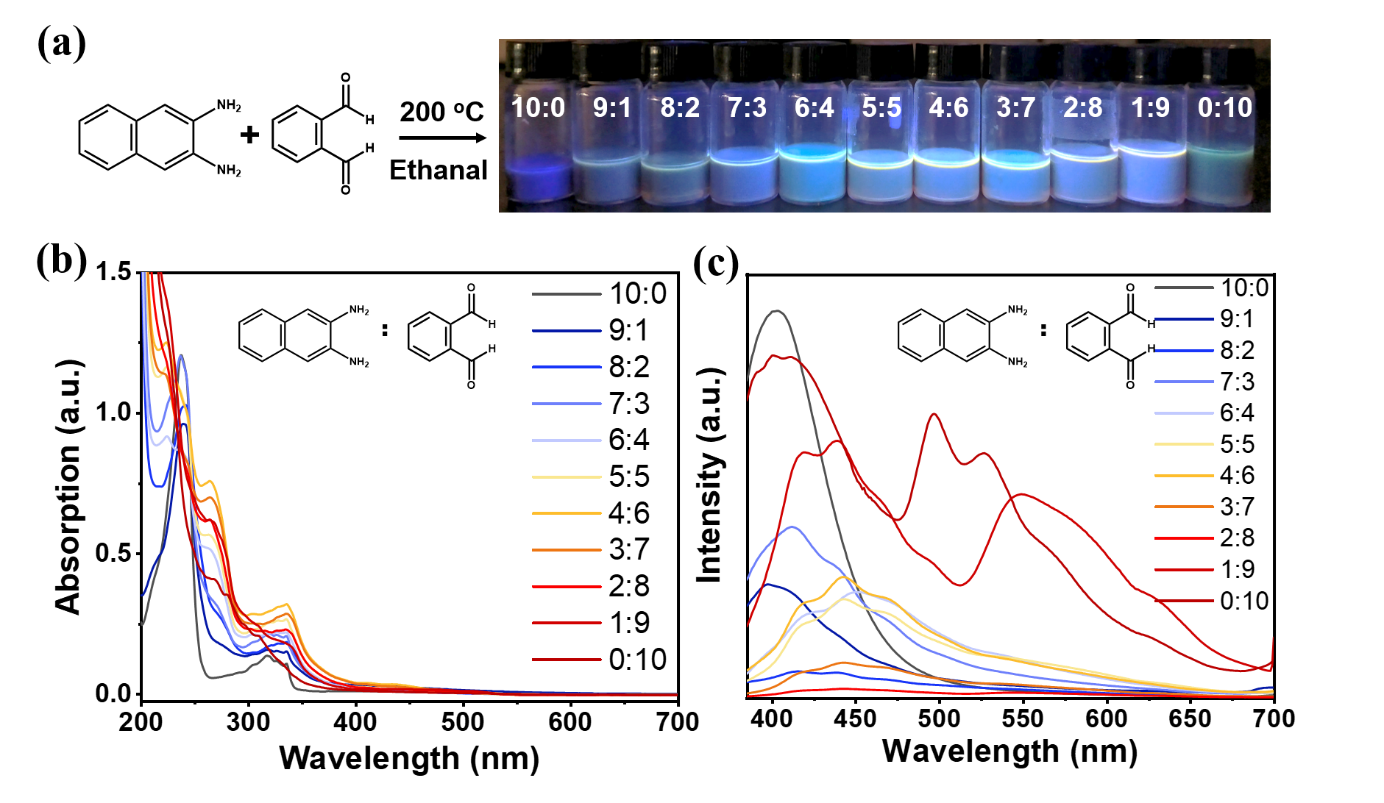
**

**Figure S1** Effect of precursor ratio on optical properties of CDs. UV−vis absorption spectra and fluorescence spectra of CDs-1, CDs-2 and CDs-3 in ethanol (*c* = 0.1 mg·mL^-1^).


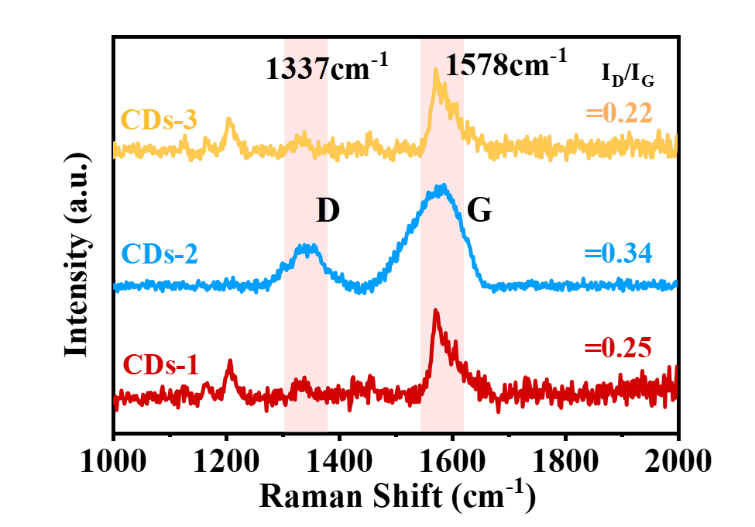


**Figure S2** Raman spectra of CDs-1, CDs-2 and CDs-3.


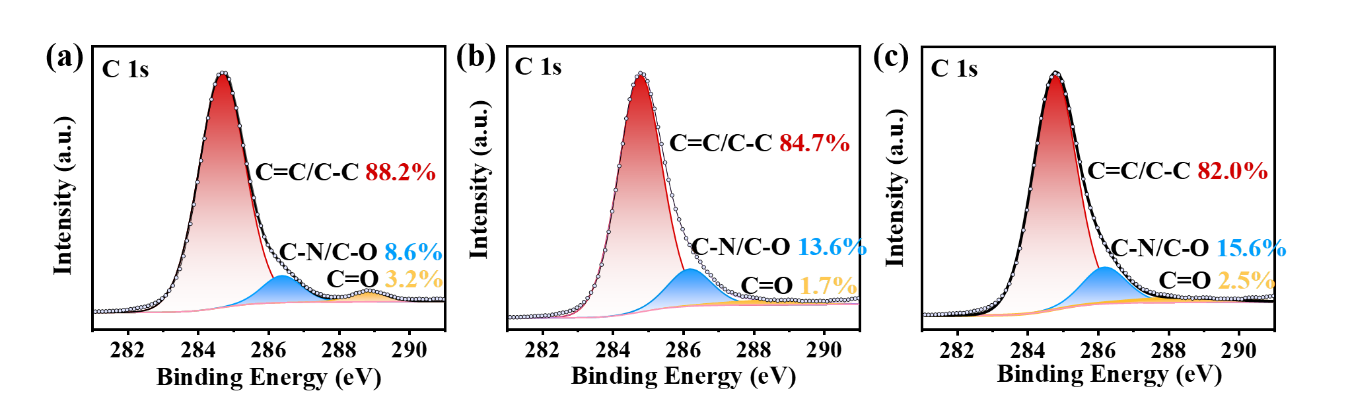


**Figure S3** O 1s peak-fitting curves of (a) CDs-1, (b) CDs-2 and (c) CDs-3.

**
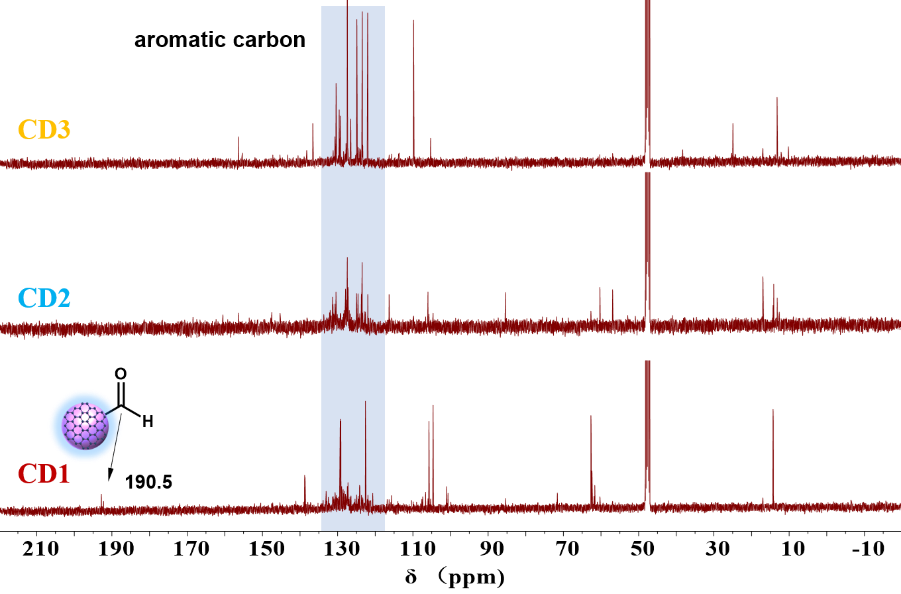
**

**Figure S4** ^13^C-NMR spectra of CDs-1, CDs-2 and CDs-3 in CD_3_OD (*c* = 50 mg·mL^-1^).


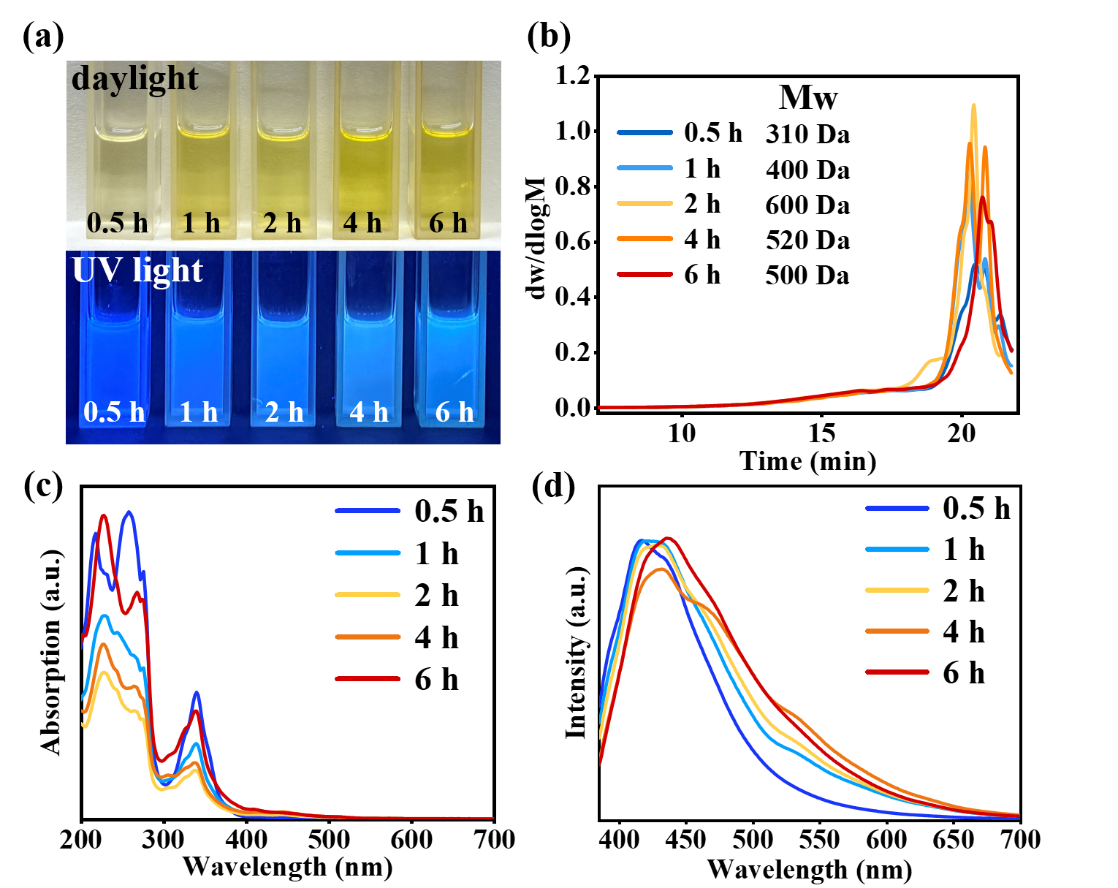


**Figure S5** Time gradient experiments for CDs-2: photographs of CDs-2 ethanol solutions exposed to (a) sunlight and ultraviolet light; (b) GPC curves showing molecular weight (Mw) evolution (ethanol, *c*=3 mg·mL^-1^); (c) absorption spectra of CDs-2 solutions; (d) fluorescence spectra of CDs-2 ethanol solutions (*c*=0.1 mg·mL^-1^).

**
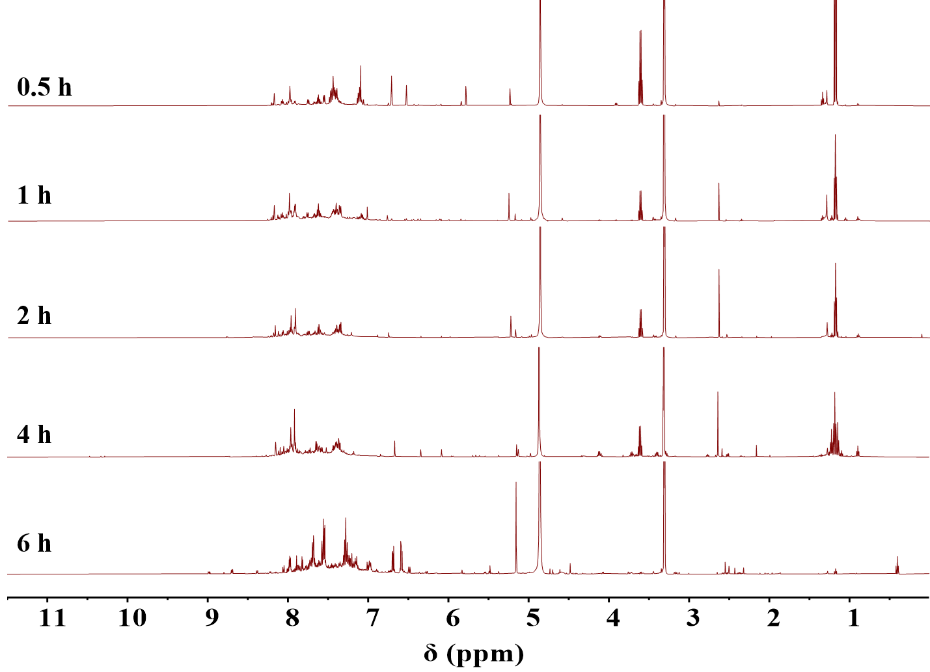
**

**Figure S6** ^1^H-NMR spectra of CDs-2 at different reaction times in CD_3_OD (*c* = 1 mg·mL^-1^).


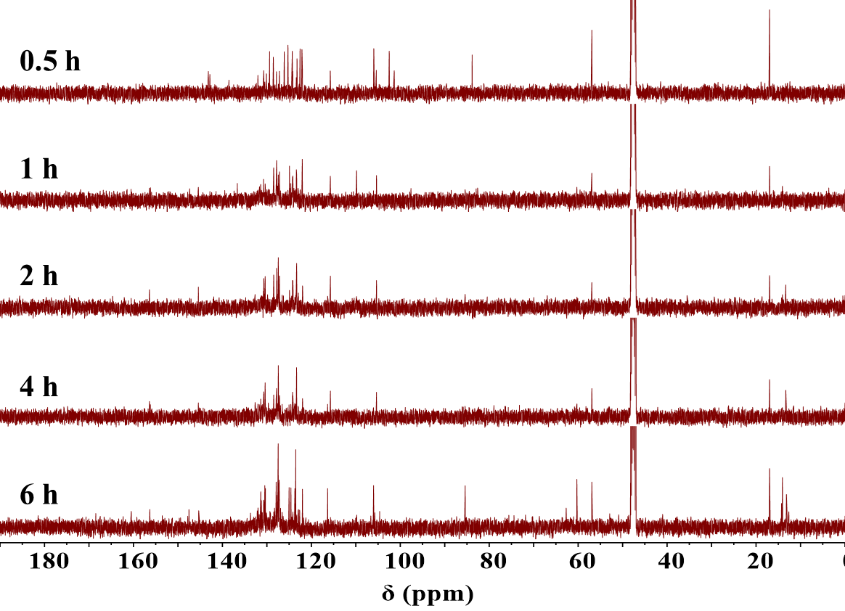


**Figure S7** ^13^C-NMR spectra of CDs-2 at different reaction times in CD_3_OD (*c* = 50 mg·mL^-1^).


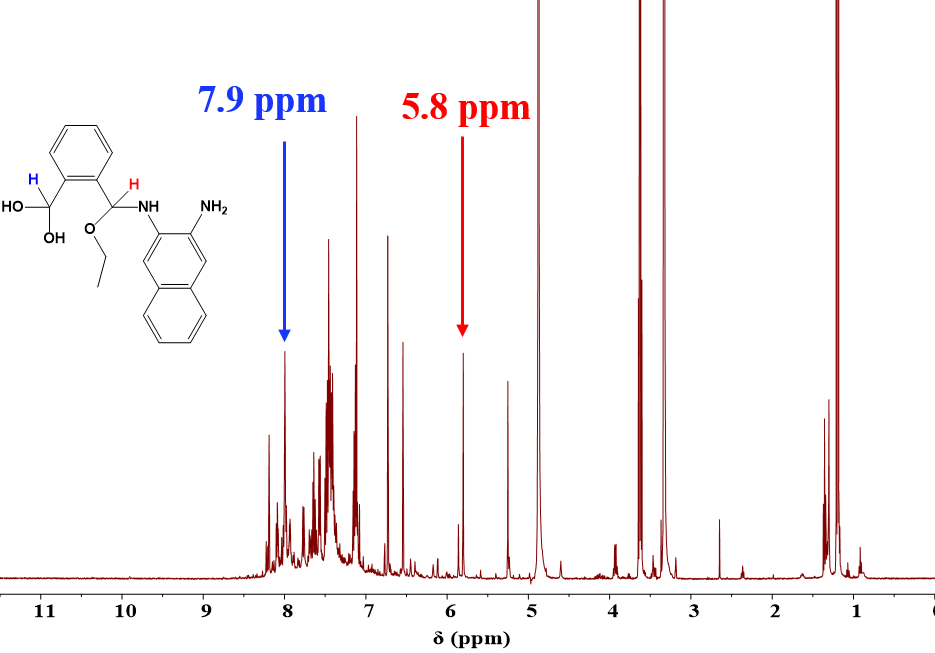


**Figure S8** ^1^H-NMR spectra of CDs-2 (10 min) in CD_3_OD (*c* = 1 mg·mL^-1^).


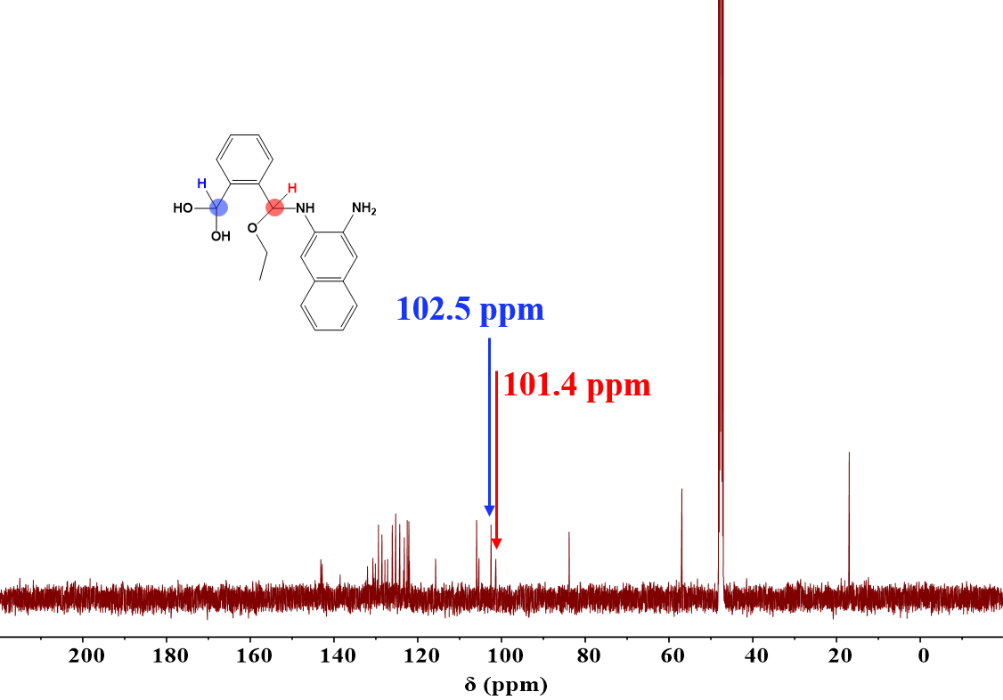


**Figure S9** ^13^C-NMR spectra of CDs-2 (10 min) in CD_3_OD (*c* = 50 mg·mL^-1^).


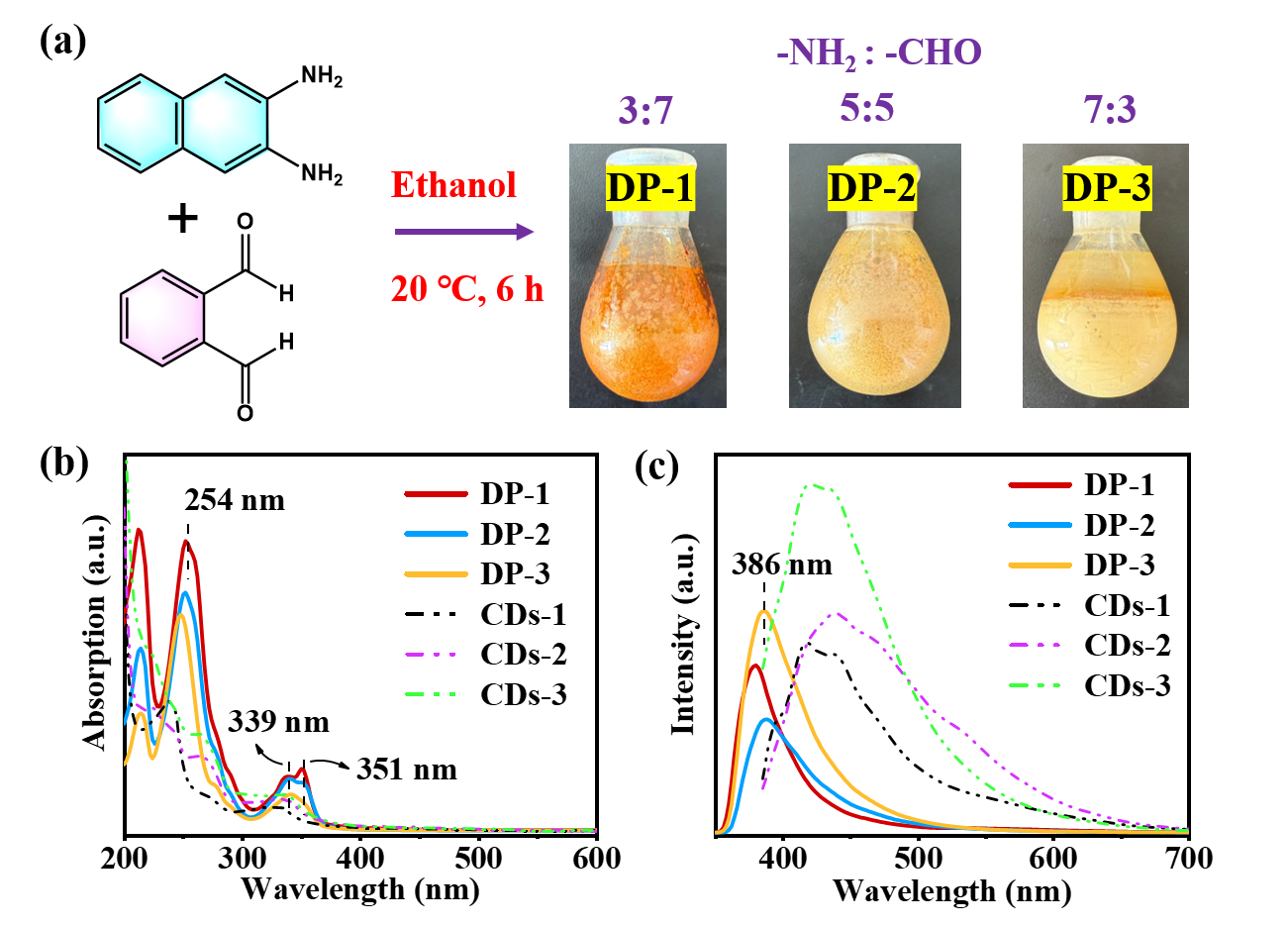


**Figure S10** (a)Schematic diagram and fluorescence photograph of the synthesis method of small-molecule analogues. The absorption spectra (b) and fluorescence spectra (c) of DP-1, DP-2 and DP-3 ethanol solutions (*c* = 0.1 mg mL^-1^) were compared with those of CDs.


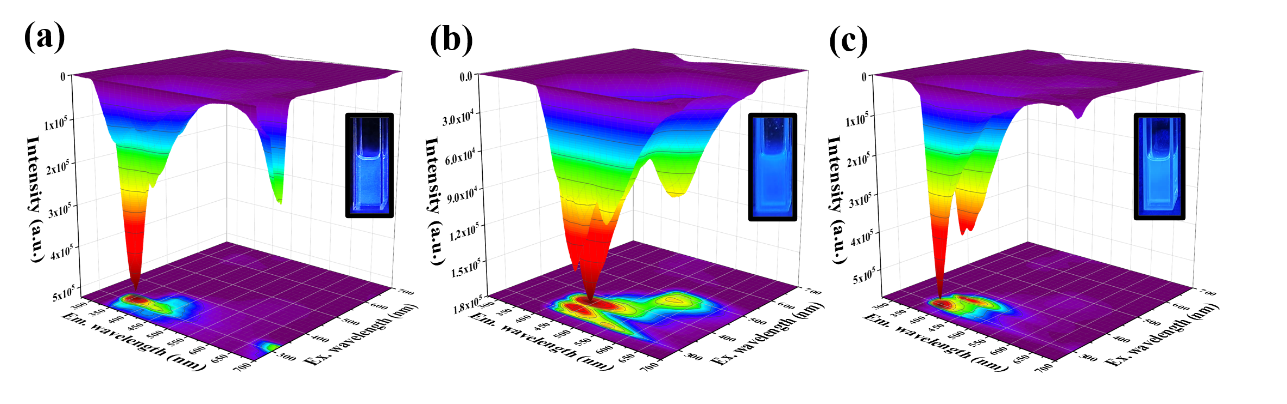


**Figure S11** 3D PL spectra of (a) CDs-1, (b) CDs-2 and (c) CDs-3 in ethanol (*c* = 0.1 mg·mL^-1^).


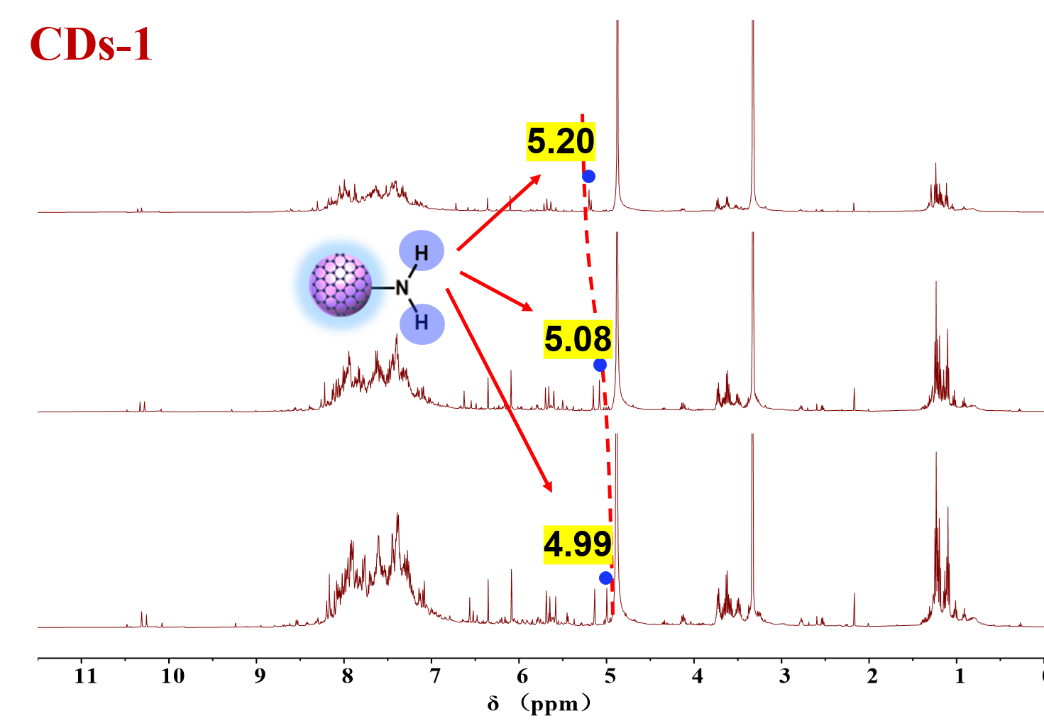


**Figure S12** ^1^H NMR spectra of CDs-1 at different concentrations (1, 5 and 10 mg·mL^-1^) in CD_3_OD.


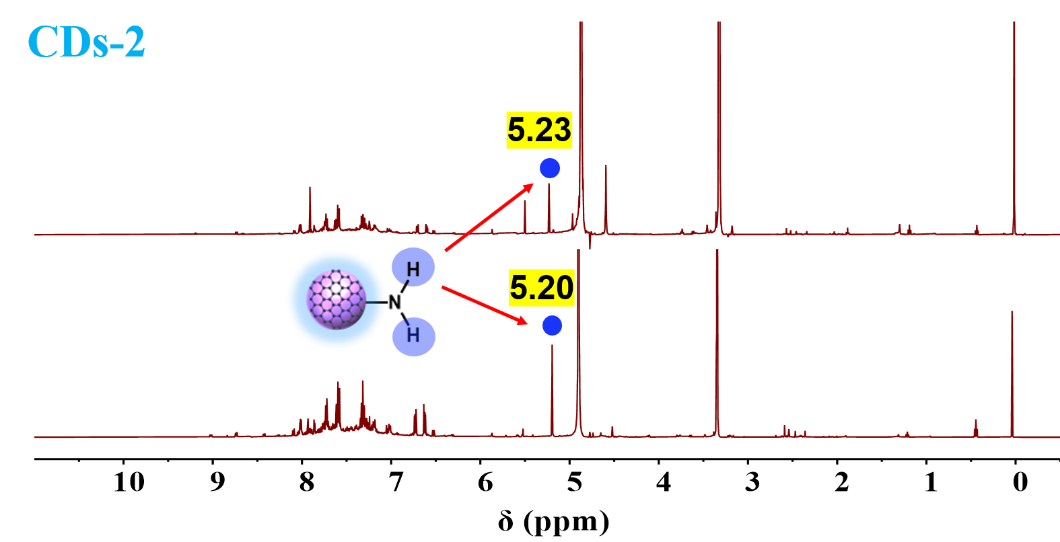


**Figure S13** ^1^H NMR spectra of CDs-2 at different concentrations (1 mg·mL^-1^ and 10 mg·mL^-1^) in CD_3_OD.


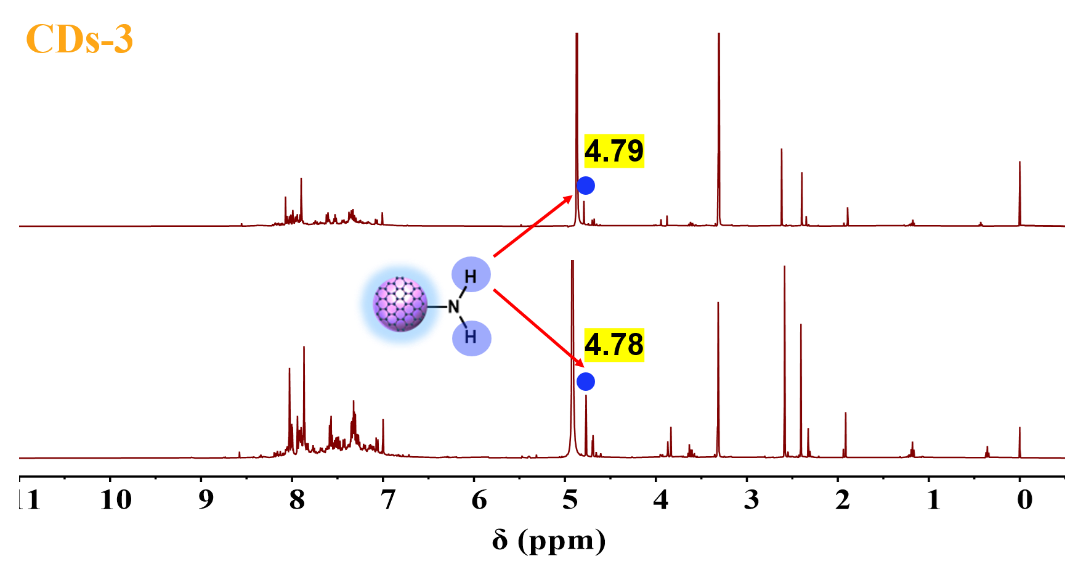


**Figure S14** ^1^H NMR spectra of CDs-3 at different concentrations (1 mg·mL^-1^ and 10 mg·mL^-1^) in CD_3_OD.


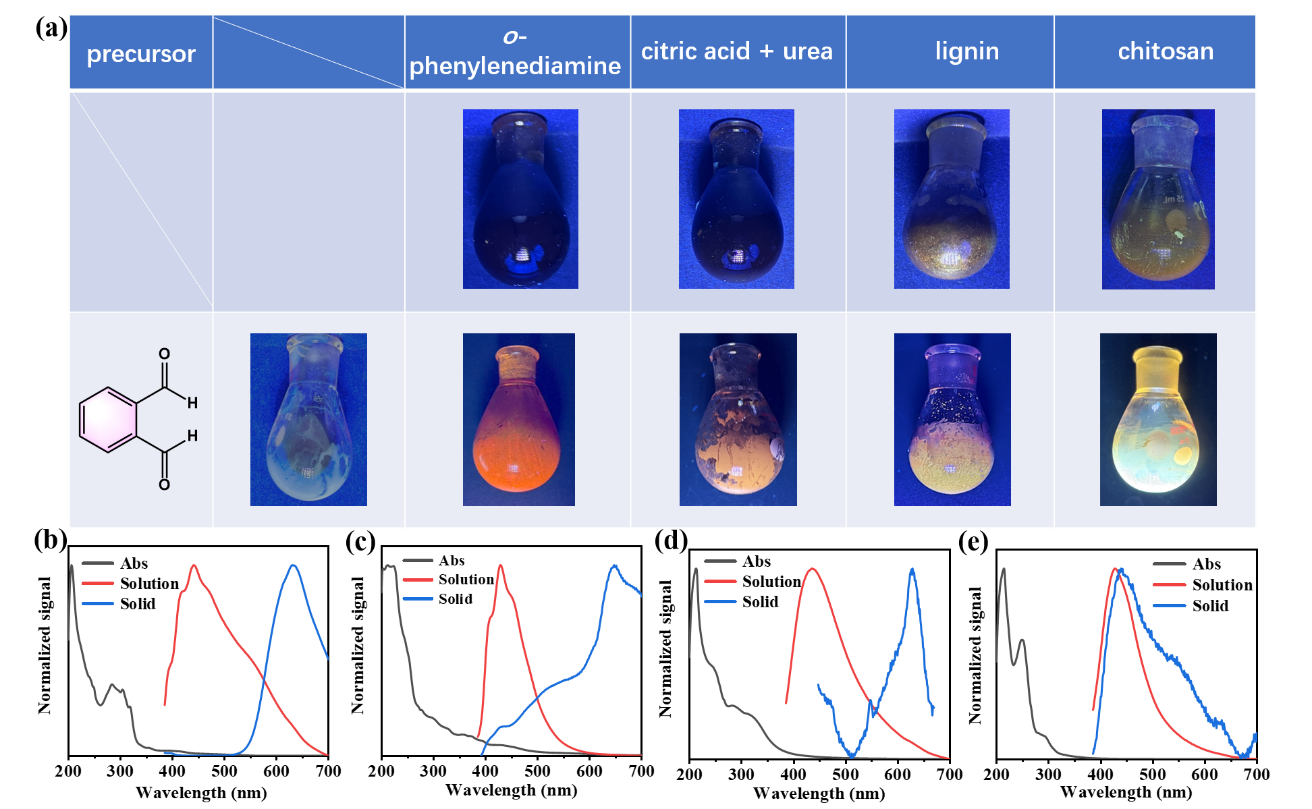


**Figure S15** (a) Images of CDs prepared with different precursor systems (under 365 nm UV-light); UV-vis absorption, solution and solid fluorescence spectra of (b) *o*-phenylenediamine, (c) citric acid and urea, (d) lignin and (e) chitosan.


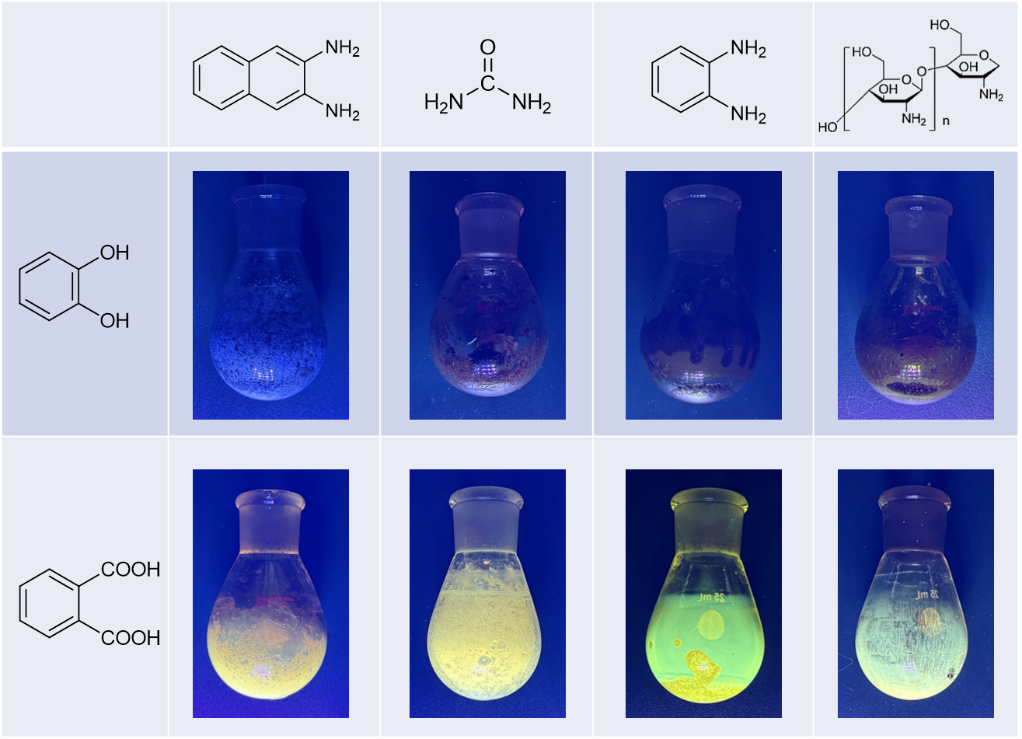


**Figure S16** Images of CDs prepared with different precursor systems (under 365 nm UV-light).


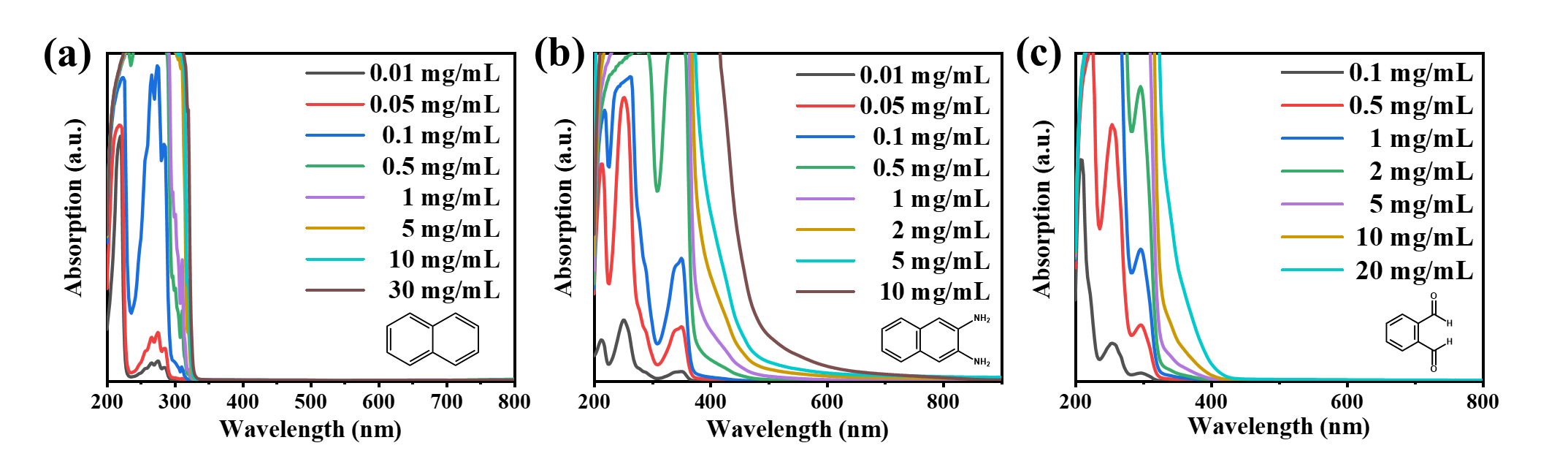


**Figure S17** Absorption spectra of (a) naphthalene, (b) 2,3-diaminonaphthalene and (c) *o*-phthalaldehyde in ethanol at different concentrations.


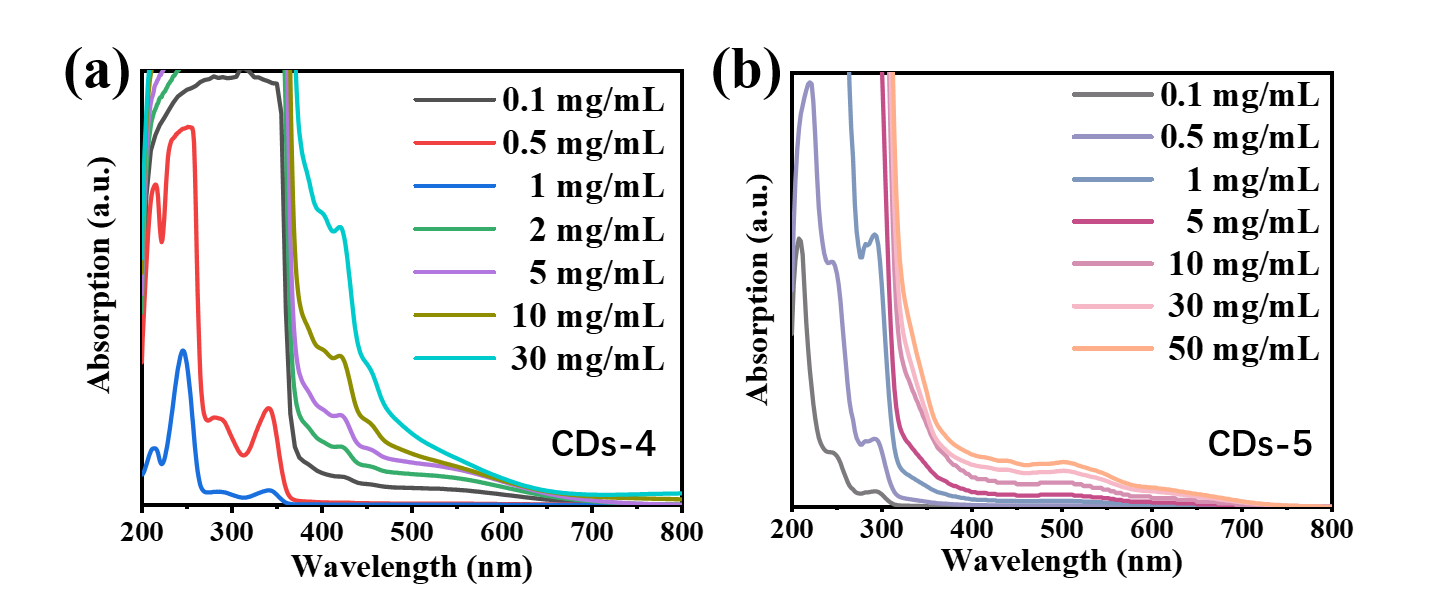


**Figure S18** Absorption spectra of (a) CDs-4 and (b) CDs-5 in ethanol at different concentrations.


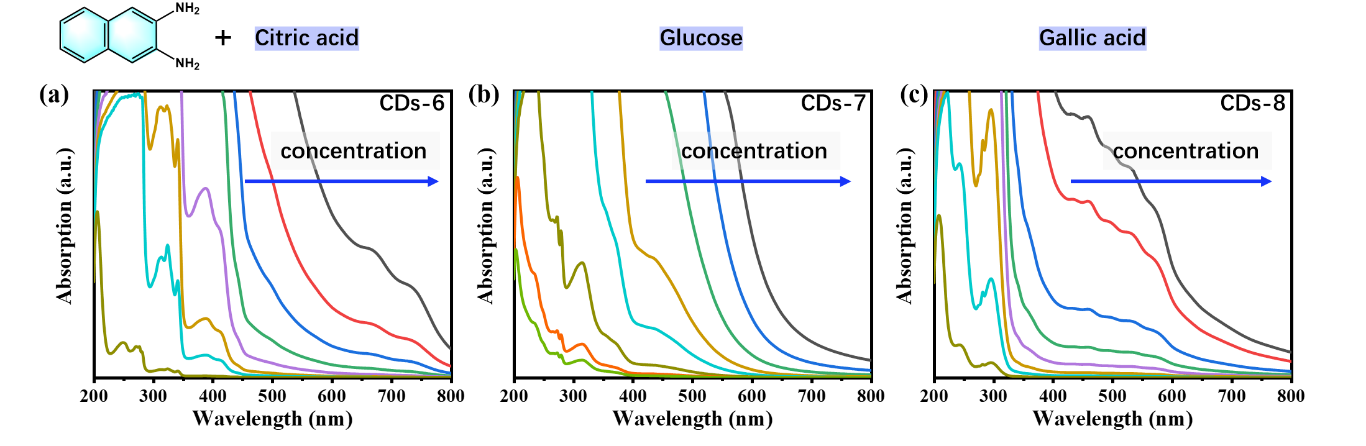


**Figure S19** Absorption spectra of (a) CDs-6， (b) CDs-7 and (c) CDs-8 in ethanol at different concentrations.


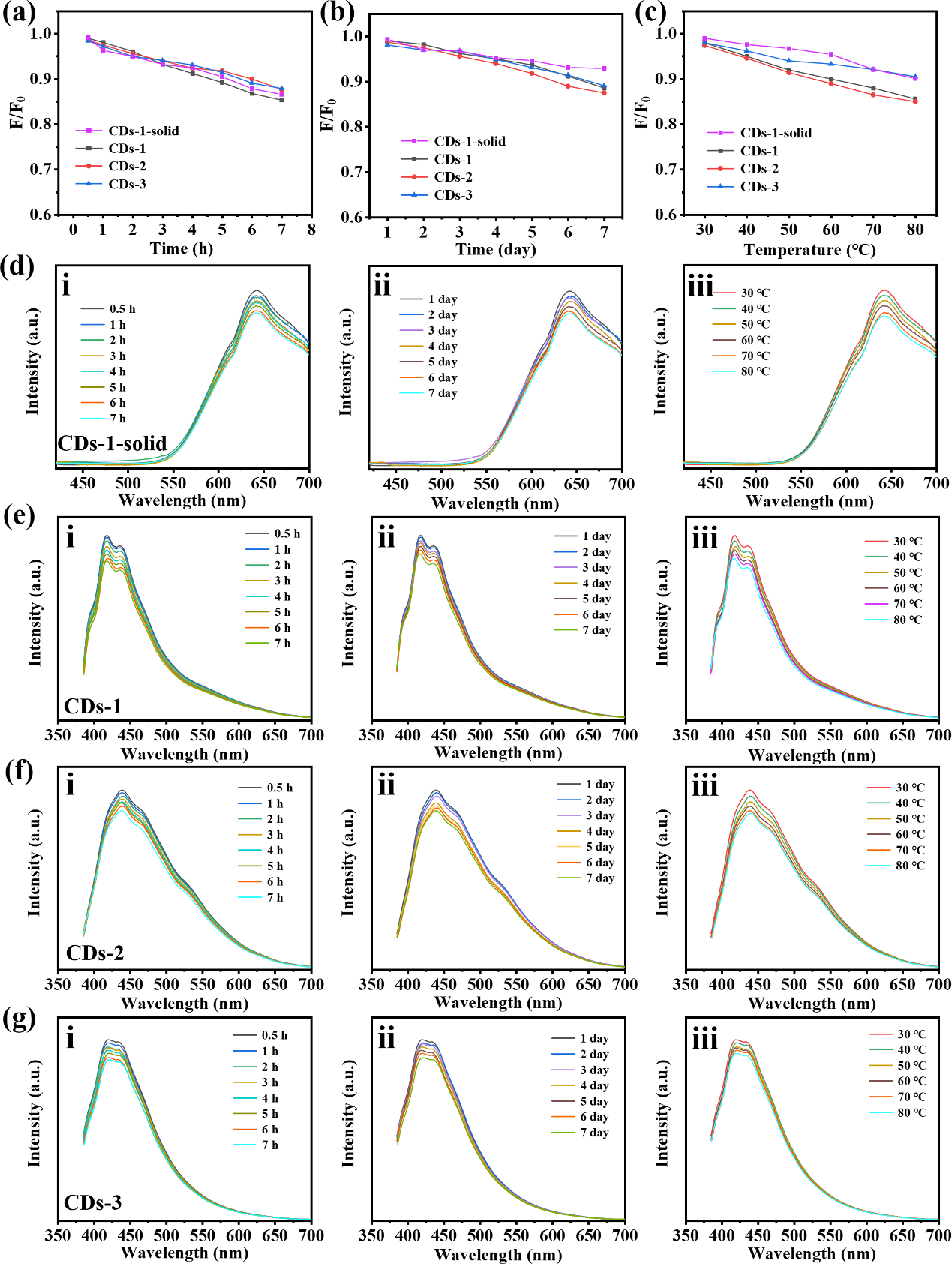


**Figure S20** Decay curve of PL intensity of CDs with UV (a), increasing visible (b) irradiation time and temperature (c).PL spectra of (d) CDs-1-solid, (e) CDs-1, (f) CDs-2 and (g) CDs-3 under different UV (i), visible (ii) irradiation time and temperature (iii).


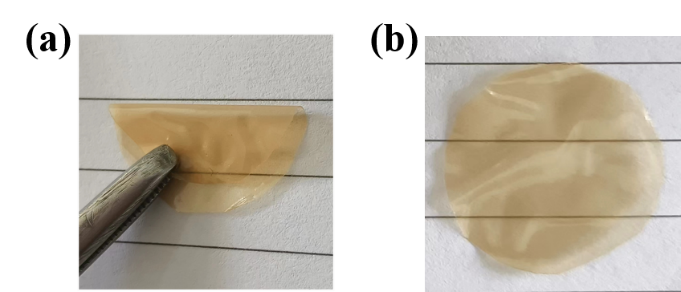


**Figure S21** (a) Flexibility of the CDs-2@HEC film. (b) Ambient atmospheric stability of the CDs-2@HEC 3 after ~1 year lapse.


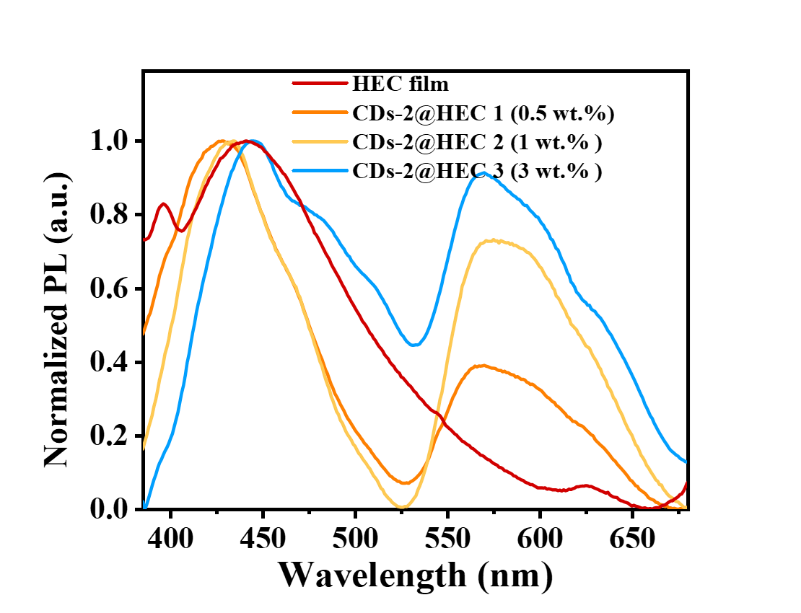


**Figure S22** FL spectra of HEC film and CDs-2@HEC films.


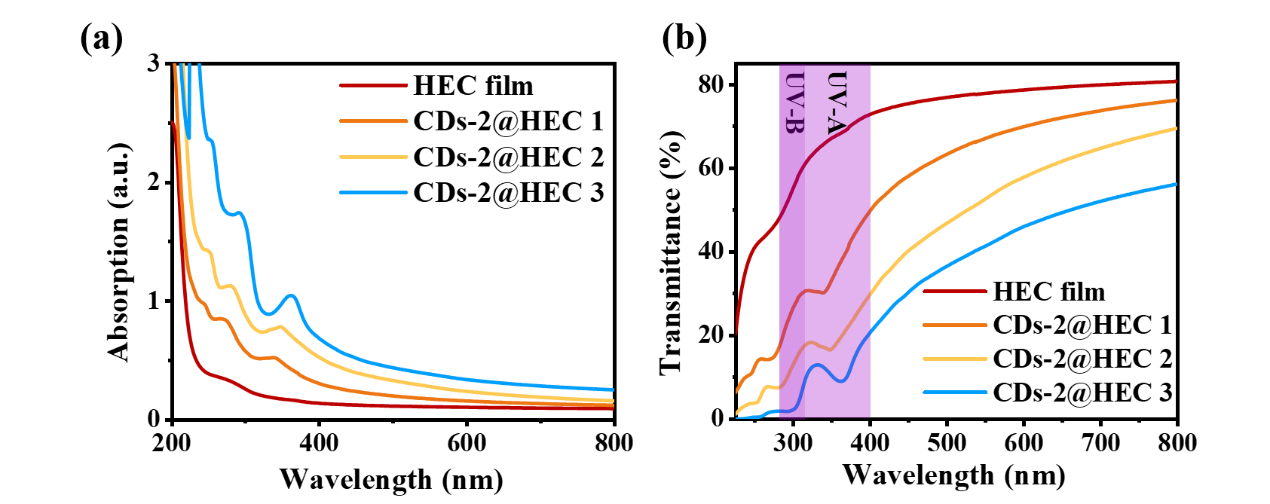


**Figure S23** (a) UV-vis and (b) transmittance spectra of different films.


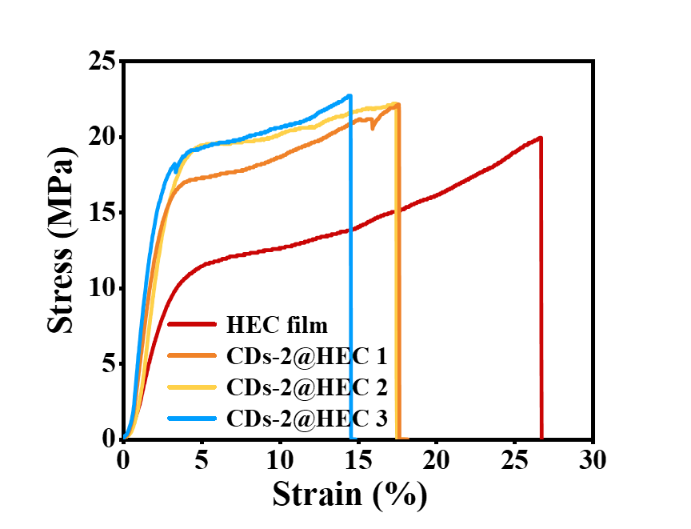


**Figure S24** The tensile stress-strain curves of the films.


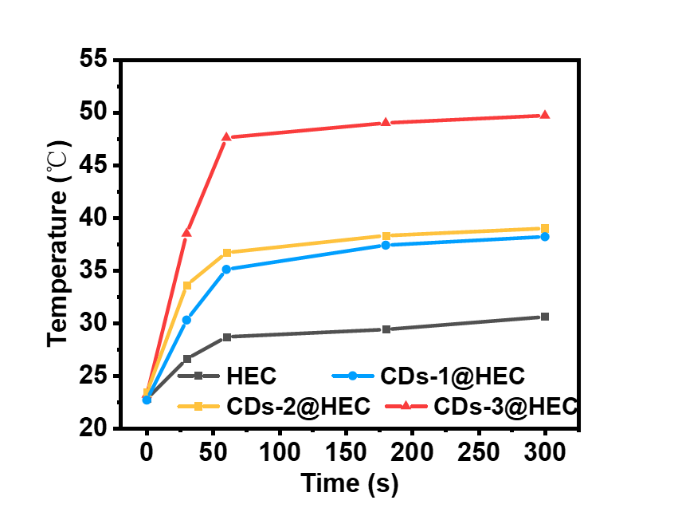


**Figure S25** Surface temperature change curves of four films in 300 s under 100 mW/cm^-2^ solar radiation.


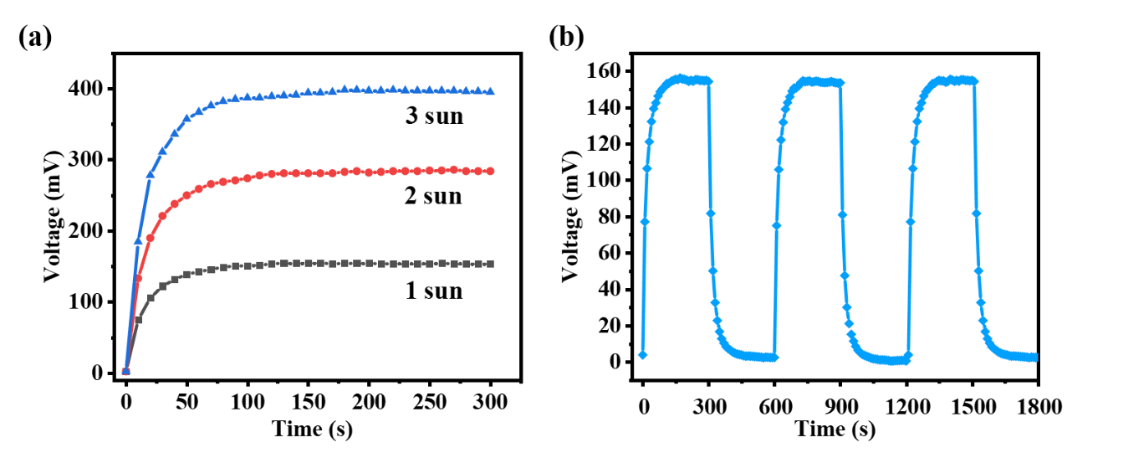


**Figure S26** (a) The voltage variation of CDs-3@HEC film under different illumination intensity; (b) The voltage change curve produced by CDs-3@HEC film at 100 mW/cm*^-^*^2^ simulating sunlight irradiation and then turning off the light source for three cycles.

**Supporting Tables**

**Table S1** Elemental proportions and chemical bonds in CDs

|  | CDs-1 | CDs-2 | CDs-3 |
| --- | --- | --- | --- |
| C 1s | 85.3% | 85.7% | 84.6% |
| O 1s | 9.4% | 6.8% | 4.4% |
| N 1s | 5.3% | 7.5% | 11.0% |
| C=C/C-C | 86.2% | 83.5% | 80.0% |
| C-O/C-N | 8.5% | 10.7% | 12.9% |
| C=O | 5.3% | 5.8% | 7.1% |
| C-O | 16.0% | 18.7% | 21.3% |
| C=O | 84.0% | 81.3% | 78.7% |
| C-N-H_2_ | 36.6% | 47.6% | 79.4% |
| C_2_-N-H | 49.5% | 37.6% | 15.9% |
| C=N-C | 13.9% | 14.8% | 4.7% |

**Table S2.** Mechanical properties of HEC film, CD2@HEC 1, CD2@HEC 2 and CD2@HEC 3.

| **Sample** | **Tensile strength (MPa)** | **Strain-at-break (%)** | **Young’s modulus (MPa)** |
| --- | --- | --- | --- |
| **HEC film** | 19.9 | 26.7 | 317.8 |
| **CD2@HEC 1** | 22.1 | 17.6 | 519.3 |
| **CD2@HEC 2** | 22.3 | 17.4 | 522.9 |
| **CD2@HEC 3** | 22.8 | 14.5 | 674.2 |
